# A drought‐responsive rice amidohydrolase is the elusive plant guanine deaminase with the potential to modulate the epigenome

**DOI:** 10.1111/ppl.13392

**Published:** 2021-04-01

**Authors:** Dhananjay Gotarkar, Toshisangba Longkumer, Naoki Yamamoto, Amrit Kaur Nanda, Tamara Iglesias, Lin‐Feng Li, Berta Miro, Elisa Blanco Gonzalez, Maria Montes Bayon, Kenneth M. Olsen, Yue‐Ie Caroline Hsing, Ajay Kohli

**Affiliations:** ^1^ Strategic Innovation Platform International Rice Research Institute Makati Philippines; ^2^ Faculty of Chemistry, Department of Physical and Analytical Chemistry University of Oviedo Oviedo Asturias Spain; ^3^ Department of Biology Washington University St. Louis Missouri USA; ^4^ Institute of Plant and Microbial Biology, Academia Sinica Taipei Taiwan

## Abstract

Drought stress in plants causes differential expression of numerous genes. One of these differentially expressed genes in rice is a specific amidohydrolase. We characterized this amidohydrolase gene on the rice chromosome 12 as the first plant *guanine deaminase* (*OsGDA1*). The biochemical activity of GDA is known from tea and coffee plants where its catalytic product, xanthine, is the precursor for theine and caffeine. However, no plant gene that is coding for GDA is known so far. Recombinant *OsGDA1* converted guanine to xanthine in vitro. Measurement of guanine and xanthine contents in the *OsGDA1* knockout (KO) line and in the wild type Tainung 67 rice plants also suggested GDA activity in vivo. The content of cellular xanthine is important because of its catabolic products allantoin, ureides, and urea which play roles in water and nitrogen stress tolerance among others. The identification of *OsGDA1* fills a critical gap in the S‐adenosyl‐methionine (SAM) to xanthine pathway. SAM is converted to S‐adenosyl‐homocysteine (SAH) and finally to xanthine. SAH is a potent inhibitor of DNA methyltransferases, the reduction of which leads to increased DNA methylation and gene silencing in Arabidopsis. We report that the *OsGDA1* KO line exhibited a decrease in SAM, SAH and adenosine and an increase in rice genome methylation. The OsGDA1 protein phylogeny combined with mutational protein destabilization analysis suggested artificial selection for null mutants, which could affect genome methylation as in the KO line. Limited information on genes that may affect epigenetics indirectly requires deeper insights into such a role and effect of purine catabolism and related genetic networks.

## 1 INTRODUCTION

Mainstream rice cultivation has been practiced in waterlogged fields for hundreds of years. This makes rice particularly sensitive to drought (Lafitte et al., 2004). Both yield and grain quality are negatively affected by water stress (Fitzgerald and Resurreccion 2009; Kim et al., 2011). Drought at the reproductive stage of the rice life cycle is particularly damaging to yield (Matsui et al., 2001; Jagadish et al., 2008). Owing to climate change and global warming, the frequency of drought has increased (Dai 2011) and those drought spells are extended in season and time, thus causing worse negative effects on agriculture, socioeconomics, and ecology (Zhang et al., 2016). Such extended drought spells can lead to up to 60% crop loss. The areas affected by drought are increasing and nearly two‐thirds of the world may encounter conditions of water stress by 2025 (Manavalan and Nguyen 2017). To deal with regular drought instances, numerous attempts have been made to identify genotypes, QTLs and genes useful for drought tolerance (Pabuayon et al., 2020). Screening for reasonable rice yield despite drought brought more success recently, and progress in identifying the relevant genotypes, QTLs and genes has led to important discoveries (Sandhu et al., 2020). An important large effect QTL for rice yield under drought (q*DTY*_*12.1*_) was identified as a regulon‐like multi‐gene QTL, (Dixit et al., 2015). Its role as a multi‐gene QTL was further substantiated through proteome and targeted metabolome analysis (Raorane et al., 2015a, b). Within the QTL gene‐cluster, various candidate genes were identified as involved in influencing yield under drought. The amidohydrolase at LOC_Os12g28270 that we report on, was one such candidate gene owing to its differential expression under water stress (Dixit et al., 2015).

In various organisms amidohydrolases form a superfamily of proteins with roles so diverse, that a separate initiative is underway to formulate strategies for assigning functionalities to members of this superfamily under the NIH Enzyme Function Initiative (EFI; https://efi.igb.illinois.edu/). This superfamily provides a good example of the divergence of protein architecture and catalytic transformations from a common ancestor. The complexity of the superfamily is evident from the fact that some amidohydrolases, whose crystal structures are available, are still not characterized for their enzymatic reactions (Seibert and Raushel 2005). Amidohydrolases catalyze the hydrolysis of substrates bearing amide, halogen, ester and other functional groups. In general many enzymes involved in the catabolism of purines and pyrimidines into urea and ammonia are amidohydrolases. In Arabidopsis an amidohydrolase‐like protein with guanosine deaminase activity catabolizes guanosine into xanthosine. (Dahncke and Witte 2013). Similarly, a guanine deaminase, which catabolizes guanine to xanthine in animal tissues and microorganisms is also an amidohydrolase (Shek et al., 2019). In plants the biochemical activity of guanine deaminases has been characterized in tea and coffee leaves where the product of guanine catabolism, xanthine, is a precursor for theine and caffeine (Negishi et al., 1994; Ashihara et al., 2008). Despite the characterization of enzymatic activity from higher plants, the gene for *guanine deaminase* is not known. This is reminiscent of the complexity of amidohydrolases for functional variability despite sequence and structure similarity (Siebert and Raushel 2005).

For a clear classification of an amidohydrolase, many substrates may need to be tested to identify the enzyme activity. The rice genome contains four loci annotated as amidohydrolases and the substrate of only one is known to be asparagine, while the other three remain uncharacterized. Here, we show that the rice amidohydrolase on chromosome 12 (LOC_Os12g28270) is a guanine deaminase (*OsGDA1*). In a knockout (KO) rice line, the upstream metabolites of the guanine catabolism pathway, particularly S‐adenosyl‐homocysteine (SAH), is reduced. The SAH reduction leads to reduced inhibition of the DNA methyltransferases, which in turn, leads to increased genomic DNA methylation. Variation in the homocysteine and SAH contents affect the expression of many genes connected to complex disorders in humans through the alteration of the DNA methylation status (Sharma et al., 2006).

The *OsGDA1* gene was cloned from both of the parental rice lines Vandana (drought tolerant) and Way Rarem (drought sensitive) involved in the original cross for the identification of a QTL for yield under drought (q*DTY*_*12.1*_). Recombinant OsGDA1 protein from the two parental alleles exhibited a clear difference in the rate of enzyme activity. We predicted the reasons for such a difference based on the DNA and protein sequence, using in silico analysis for the effect of specific mutations on protein stability and function. The comparison of the OsGDA1 protein sequence for amino acid mutations in multiple rice accessions identified various mutations with an effect on protein stability. Depending on the kind of mutations, the protein sequences could be classified into specific clades, which suggested evolutionary processes at play for the kind and extent of variation in the OsGDA1 protein. We discuss the importance of this variability for possible roles in rice plant growth, development and stress response.

## 2 MATERIALS AND METHODS

### 2.1 Cloning and characterization of OsGDA1 recombinant proteins of Way Rarem and Vandana

For *OsGDA1* characterization, the rice cultivars Vandana and Way Rarem described earlier (Bernier et al., 2007; Dixit et al., 2015) were grown in conditions similar to those described by Henry et al. (2014). Leaves from booting stage plants were used for amplification of cDNA of Way Rarem and Vandana *OsGDA1* using primers AMI_pGEX_F and AMI_pGEX_R (Table S6) and cloned into the pGEX 4T1 vector (GE healthcare) with a BamHI and XhoI fragment. The vector was transformed into *E.coli BL21* cells and recombinant protein was induced using 1 mM IPTG.

### 2.2 GST‐tagged recombinant protein sample preparation, purification and quantification

All the operations in this step were performed either on ice or at 4°C. The pellet of recombinant *E.coli BL21* cells was stored in −80°C after induction with 1 mM IPTG, was suspended in 7 ml of binding buffer per g of pellet (140 mM NaCl, 2.7 mM KCl, 10 mM Na_2_HPO_4_, 1.8 mM KH_2_PO_4_, pH 7.3), and gently vortexed to lyse the cells until it formed a homogeneous solution, followed by sonication at 45 s ON, 45 s OFF for 10 cycles at 10 microns amplitude. Further, the homogeneous solution was centrifuged at 7740*g* for 10 min at 4°C to separate the cell debris and cellular components including soluble proteins and inclusion bodies. The obtained supernatant was centrifuged at 17 400*g* for 30 min at 4°C which resulted in soluble proteins and inclusion bodies in the supernatant and pellet, respectively. We added 2 ml ST buffer (50 mM Tris, 300 mM NaCl, 5 mM ZnCl_2_, 10 mM β‐mercaptoethanol with 10% [w/v] sarkosyl) to the pellet with the inclusion bodies and stored at 4°C overnight to solubilize the recombinant protein from the inclusion bodies (Tao et al., 2010). After the overnight incubation, all the tubes with ST buffer were diluted 10x with binding buffer to dilute the concentration to 1% Sarkosyl, and centrifuged at 12000 rpm for 10 min at 4°C to separate cell debris and proteins. The supernatant was combined with the soluble protein fraction (2nd supernatant after sonication) and with 2% Triton‐X100 and 20 mM CHAPS and mixed with gentle shaking. This solution of proteins and reagents was used as samples to inject to glutathione sepharose column at the rate of 2 ml min^−1^ to separate the GST tagged recombinant Way Rarem and Vandana OsGDA1 proteins in different batches. The instructions in the manual for Glutathione Sepharose 4 Fast Flow were followed (GE Healthcare Biosciences). The vector encoded GST protein was purified and used as negative control for enzymatic assays. The purified GST tagged recombinant protein fraction was checked for purity on SDS PAGE and quantified using the Bradford method (Kruger, 2009) before being used for enzyme activity tests.

### 2.3 Determination of enzyme activity kinetics using HPLC

The quantified recombinant Way Rarem and Vandana proteins were used for enzyme assay with 10 different substrates, which were as follows: Adenine, Adenosine, 2′‐Deoxyadenosine, 5′‐Deoxyadenosine, 5′‐Deoxy‐5′‐(methylthio)adenosine, Guanine, Guanosine, S‐adenosyl homocysteine, Xanthosine, and Atrazine. Five hundred nanograms of recombinant protein was incubated with 150 mM of each substrate at 30°C in dark for 3, 6, and 9 h and overnight in 1 ml 100 mM sodium phosphate buffer, pH 7.4, and the reaction was terminated adding 50 μl of 30% perchloric acid. The reaction mixture was filtered through a 0.2 μm syringe filter and the separation was done on the C18 reverse‐phase column (250 mm × 4.6 mm, Atlantis dC18, 5 μm) by following the HPLC method as described in (Wang et al., 2001). The mobile phase consisted of two solvents: Solvent A, 8 mM octanesulfonic acid sodium salt and 50 mM NaH_2_PO_4_ adjusted to pH 3.0 with H_3_PO_4_ and Solvent B, 100% methanol. Before use, solvent A was filtered through a 0.2 mm membrane filter. The HPLC column was equilibrated with 80% Solvent A and 20% Solvent B. The sample was injected and separation was obtained using a step gradient. The gradient consisted of 8 min at the equilibration conditions, 30 sek to increase Solvent B to 40%, 12.5 min at the new condition, and 30 sek to return to the equilibration conditions and a minimum of 10 min before a subsequent injection. The flow‐rate was 1 ml min^−1^ and detection was monitored at 254 nm. The HPLC was performed at room temperature. The enzyme activity rate was calculated based on the substrate consumed in relation to incubation time (Pace et al., 1989).

### 2.4 OsGDA1 protein destabilization and phylogeny analysis

All the cultivated rice accessions were selected from the 3 K data based on the coverage and geographic distributions. Only high coverage (greater than 15x) cultivated accessions were chosen. Similarly, the wild accessions (Huang et al., 2012) were downloaded according to their genome coverage and geographic distributions. Raw data were analyzed after downloading and by assembling the short reads using BWA (Li and Durbin, 2010) and the SNPs were reported using SAMtools (Li et al., 2009). The quality of raw reads was evaluated using FastQC (Schmieder and Edwards, 2011) and the low‐quality (Phred <30) reads were removed. Alignments of the clean reads were initially screened against the Nipponbare reference (MSU6) using Burrows‐Wheeler Aligner (Li and Durbin, 2010). A neighbor‐Joining tree of both wild and cultivated rice protein sequences was constructed (Saitou and Nei, 1987). The percentage of replicate trees in which the associated accessions clustered together in the bootstrap test (1000 replicates) was shown next to the branches (Felsenstein, 1985). The tree was drawn to scale, with branch lengths in the same units as those of the evolutionary distances used to infer the phylogenetic tree. Evolutionary distances were computed using the Poisson correction method (Zuckerkandl and Pauling, 1965) and were in units of the number of amino acid substitutions per site. The analysis involved 472 amino acid sequences. All positions containing gaps and missing data were excluded. Evolutionary analyses were conducted in MEGA7 (Kumar et al., 2016). Clades were shown with numbers 1, 2, 3, etc. Protein amino acid changes and prediction of functional consequences were analyzed with Variant Effect Predictor tool (McLaren et al., 2016). Mutation stability was calculated using the EASE‐MMsoftware (Folkman et al., 2016) and the DIM‐Pred software (Anoosha et al., 2015) http://www.iitm.ac.in/bioinfo/DIM_Pred/reference.html. Protein post‐translation modification at sites for amino acid alterations was performed as per Motif scan tool (Pagni et al., 2007; http://myhits.isb-sib.ch/cgi-bin/motif_scan).

### 2.5 Plant material for estimation of DNA methylation and metabolite quantification

Tainung 67 (T67), wild type (WT), and the *OsGDA1* homozygous knockout mutant (KO), were obtained from the Taiwan Rice Insertional Mutants (TRIM) Database, Academia Sinica, Taiwan. Three biological replicates each of KO and WT were used in the experiments. The leaf and root genomic DNA of 21 days old seedlings grown in the glasshouse was extracted using the CTAB method (Allen et al., 2006) and was used in all the approaches for qualitative and quantitative genomic DNA methylation analysis.

### 2.6 Densitometric analysis

The *Msp*I*/Hpa*II isoschizomer digest was carried out on 3 μg of genomic DNA in a 20 μl volume for 16 h and the digestion product was run on an 0.8% agarose gel to visualize the digestion pattern. Densitometry analysis was done using the quantity one software (Biorad) and the volume was calculated by multiplying optical density and area of the band selected. The portion to be analyzed was selected at the top part of the gel in the area marked in Figure S8, and the area was kept constant for all the selected portions of the bands.

### 2.7 Methyl‐sensitive amplification polymorphism (MSAP) approach

Methylation‐sensitive amplification polymorphism (MSAP) was performed according to the protocol described by Xu et al. (2013). The subsequent electrophoresis of PCR amplified product was run on an denaturing urea PAGE at 50 volts for 12 h at 25°C and was silver stained for visualization. Differential banding pattern between genotypes was scored and analyzed by formula as described by Xu et al. (2013).

### 2.8 ELISA‐based global genomic DNA methylation quantification

Global DNA methylation was quantified as the percentage relative to control methylated DNA by using the anti‐5mC antibody from Imprint^@^ Methylated DNA Quantification Kit as per manufactures instructions manual supplied with the kit (Sigma–Aldrich; Catalog number—MDQ1). Three biological and three technical replicates for each sample were used. The environmental error which may occur without enough replicates was normalized. To calculate methylation levels relative to the control methylated DNA sample, the following formula was applied: [(*A*_450 av_ Sample − *A*_450 av_ Blank)/(*A*_450 av_ Methylated Control DNA − *A*_450 av_ Blank)] × 100 where the A450 av represent the averages of the blank, the sample and the methylated control DNA replicates.

### 2.9 HPLC‐based DNA methylation quantification

One microgram genomic DNA, from the seedlings of each line was lyophilized using the Christ Alpha 2–4 LSC lyophilizer for 24 h (Martin Christ Gefriertrocknungsa, Germany). The lyophilized samples were shipped on dry ice to the Department of Physical and Analytical Chemistry, University of Oviedo, Asturias in Spain for methylation analysis. For quantification, genomic DNA methylation was determined using the HPLC Agilent 1100 series system (Agilent Technologies) consisting of a four‐channel on‐line degasser, a standard binary pump, a micro‐well plate autosampler, and a photodiode array detector. The chromatographic separation was achieved at room temperature using a Mono‐Q HR 5/50 GL (50 × 5.0 mm, 10 μm particle size) from GE Healthcare (GE‐Healthcare Bio‐Sciences AB) at a flow rate of 1.0 ml min^−1^. The mobile phases consisted of water containing 1 mM ammonium hydroxide (phase A) and 1 M ammonium acetate also containing 1 mM ammonium hydroxide pH 6.9 (phase B). The injection volume was 20 μl. Elution was performed with a gradient from 8 to 50% B in 30 min. DNA samples were reconstituted in 100 μl of water and treated with RNase (Thermo Fisher Scientific) in order to remove some RNA still present in the samples that was interfering in the chromatographic separation. For this aim, 2 μl of 1 μg μl^−1^ RNase was added to each of the samples and heated for 30 min at 37°C. Finally, the enzyme was removed by using the Illustra GFX PCR DNA and Gel Band Purification Kit (GE Healthcare). Then, 10 μl of Nuclease S1 from *Aspergillus oryzae* (Sigma–Aldrich; solution containing 30 mM sodium acetate, 50 mM NaCl, 1 mM ZnCl_2_, 50% glycerol and 2 mg ml^−1^ protein) was added, and the mixtures were incubated for 14 h at 37°C. The enzyme was then separated out of the suspension by membrane ultracentrifugation using Centricon YM‐10 (Merck Millipore) and the samples were directly analyzed by HPLC‐UV (270 nm).

### 2.10 LC–MS/MS‐mediated analysis of SAM and SAH

The determination of SAM and SAH was performed using an Agilent 1290 HPLC system coupled to an Agilent 6460 triple quadrupole mass spectrometer (Agilent Technologies) equipped with a jet stream electrospray ion source. Chromatographic separation was achieved at room temperature using a Zorbax Eclipse Plus C18 column Rapid Resolution HD (50 × 2.1 mm, 1.8 μm) from Agilent Technologies at a flow rate of 0.25 ml min^−1^. The mobile phases consisted of water containing 5 mM ammonium acetate (phase A) and acetonitrile also containing 5 mM ammonium acetate (phase B). The injection volume was 2 μl. Elution was performed with a gradient starting at 2% B for 1.0 min, increasing to 15% in 5.0 min. Phase B was then increased to 90% within 2.0 min and kept at this value for one more minute before it was decreased to initial conditions. The overall runtime was 12 min. MS analysis was performed in the positive‐ion mode using the multiple reaction monitoring (MRM). The capillary voltage was set at 3500 V and nozzle voltage at 0 V. The nitrogen drying gas flow was 5 l min^−1^ at a gas temperature of 350°C. The nebulizer pressure was set at 45 psi with a sheath gas flow rate of 11 l min^−1^ at temperature of 375°C. A calibration curve was obtained between 0 and 100 ng ml^−1^ for SAM (*y* = 12.3*x* − 10.7, *R* ^2^ = 0.999) and for SAH (*y* = 8.48*x* − 1.37, *R* ^2^ = 0.999). The lyophilized samples were reconstituted in 200 μl of water and then ultrafiltered using a Centricon YM‐3 (Merck Millipore). Samples were diluted 1:20 to minimize matrix effects right before injection. Peak areas were obtained by integration using Origin 8.0.

### 2.11 LC‐q‐TOF‐MS analysis of guanine, adenine, and xanthine

The quantitative analysis of guanine, adenine, and xanthine was conducted by coupling reversed phase high‐performance liquid chromatography (HPLC, UltiMate 3000, Thermo Scientific) in a Zorbax Eclipse Plus C18 column using a linear gradient of acetonitrile buffered with ammonium acetate (pH 4) coupled to a quadrupole‐time‐of‐flight mass spectrometer using electrospray ionization (ESI‐Q‐TOF‐MS, impact II, Bruker). Monitored masses were the corresponding protonated molecular ions [M + H^+^] for adenine (m/z 136.06177), guanine (m/z 152. 05669) and xanthine (m/z 153.04070). Linear calibration curves from 0 to 1000 ng ml^−1^ were obtained with 7–10 calibration points.

### 2.12 QRT‐PCR analysis

Total RNA was isolated from leaves of WT, KO, Way Rarem, and Vandana 21 days old seedlings grown in glasshouse conditions, using TRIzol reagent (Invitrogen) according to the manufacturer's instructions. The RNA was treated with 2 μl of RNase‐free DNAseI and buffer from Promega. The solution was allowed to stand at 25°C for 15 min before adding 2 μl of DNAse stop solution. The reaction was stopped at 65°C for 10 min. The RNA concentration was determined using a NanoDrop spectrophotometer. First strand cDNA was synthesized using ImProm‐IITM Reverse Transcription System (Promega). A 10 μl reaction volume consisted of 1.0 μl of normalized cDNA, 5 μl of ×2 SYBR green PCR master mix (Roche Diagnostics GmbH) and 0.4 μl of 10 mM primer for each primer pair. Reactions were run in triplicate, with biological replicates when necessary, in a 7500 Fast Real‐Time PCR System (Applied 5144 Biosystems). Amplification conditions were 50°C for 2 min, 95°C for 2 min, 40 cycles of denaturing at 95°C for 10 s, and a combined annealing and extension step at 60°C for 30 s, followed by a disassociation stage from 60 to 95°C (melting curve analysis). Primers used are listed in Table S6.

### 2.13 Western blotting

Total soluble proteins from flag leaf and roots of WT and KO plants were extracted using the TCA‐Acetone method (Niu et al., 2018) and resolved on a 10% SDS‐PAGE. The extracted protein was then transferred to a PVDF membrane (GE healthcare) and a western blot was performed using a polyclonal antibody generated using OsGDA1 specific peptides CDLYEKHHNTADGRI and CMREKKIVNLNEEEV (GeneScript). HRP conjugated secondary antibody was used and the signal was detected using Novex ECL Chemiluminescent Substrate Reagent Kit (Thermo Fisher Scientific) by exposing to an X‐ray film (GE healthcare).

## 3 RESULTS AND DISCUSSION

### 3.1 The rice amidohydrolase on chromosome 12 is a guanine deaminase

The nucleotide sequence of the LOC_Os12g28270 amidohydrolase was highly (98–85%) similar to other amidohydrolases or uncharacterized plant proteins in rice, maize and Vitis. The closest similarity to functionally characterized genes was 86.74% to the *Ricinus atrazine* chlorohydrolase and 70% to the Clostridium S‐adenosyl homocysteine deaminase (SAHD). The predicted protein sequence BLAST for LOC_Os12g28270 also revealed high similarity (91–86%) to SAHD in the *Oryza*, *Panicum*, *Setaria*, *Zea*, and *Triticum* genus. Deamination of S‐adenosyl homocysteine (SAH) converts SAH into S‐inosine homocysteine (SIH). SAH is a potent inhibitor of DNA methyltransferases (DMTs; James et al., 2002) but SIH does not inhibit DMTs (Zappia et al., 1969). There was also high similarity (73%) to the mammalian and bacterial guanine deaminase (GDA) with critical identity in substrate binding and catalytic sites.

**FIGURE 1:**
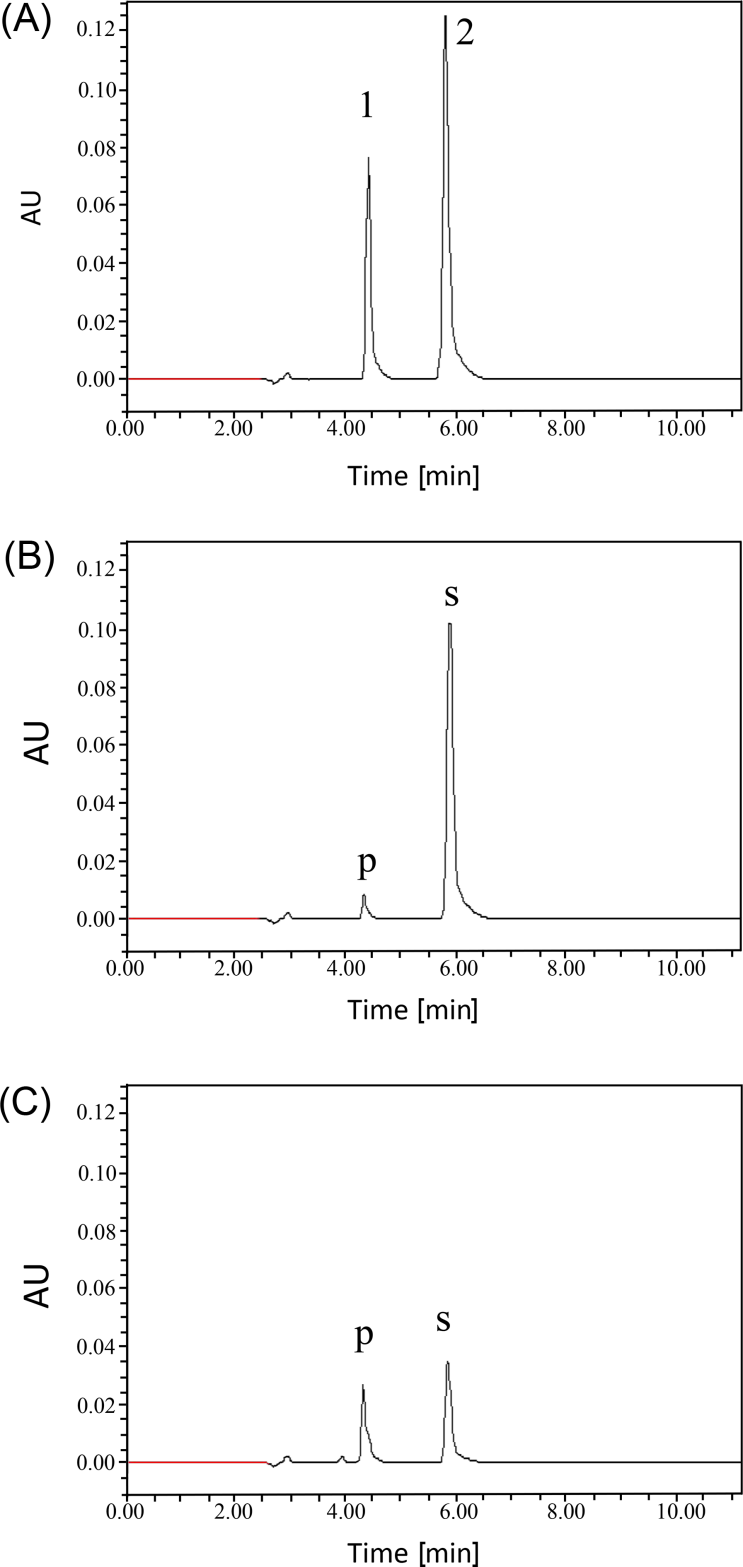
In vitro assay of recombinant OsGDA1 with guanine. (A) HPLC elution peak for the standards (1) xanthine and (2) guanine. (B, C) Activity of the recombinant OsGDA1 of Vandana and Way Rarem, respectively. More of the guanine substrate (s) is utilized and more of the xanthine product (p) is produced by the recombinant protein from Way Rarem (C), when all other variables are constant in the assay. X axis represents time in minutes and Y axis represents the arbitrary units (AU). Even if the Vandana protein was increased, the activity was lower than Way Rarem

Amidohydrolases are mostly involved in nucleotide metabolism (Holm and Sander, 1997) especially for cytosine and adenine. Not much is known about amidohydrolases active in thymine and guanine metabolism. The amidohydrolase gene identified here was thus cloned from the two rice accessions, Vandana (drought tolerant) and Way Rarem (drought susceptible), used in the primary cross that led to the identification of the QTL q*DTY*_*12.1*_ for rice yield under drought by Bernier et al., 2007. The GST‐tagged recombinant proteins were purified for both the accessions (Figure S1). Enzyme assays for *SAHDH* were negative on repeated and modified attempts. Nevertheless, the HPLC‐based *GDA* assay of the purified recombinant proteins was successful with guanine as the substrate and xanthine as the expected product. The recombinant GDA protein from the drought susceptible line Way Rarem was more active than the recombinant GDA protein from the drought tolerant line Vandana (Figure 1). It must be mentioned that q*DTY*_*12.1*_ uniquely originated from the susceptible parent of the cross, i.e. Way Rarem (Bernier et al., 2007; Dixit et al., 2015).

Among the additional metabolites tested as substrates for OsGDA1, the results were negative for Adenine, Adenosine, 5′‐Deoxyadenosine, 5′‐Deoxy‐5′‐(methylthio)adenosine, Guanosine, S‐adenosyl homocysteine, Xanthosine, and Atrazine. However, 2′‐deoxyadenosine was utilized as a substrate by both recombinant proteins (Table S1). Despite the possibility of an additional substrate, a higher rate of activity for guanine deamination (Figure S2) favored the protein coded by LOC_Os12g28270 as GDA. These results indicated that the recombinant proteins function largely as GDA and less as a 2′‐deoxyadenosine deaminase in vitro.

**FIGURE 2:**
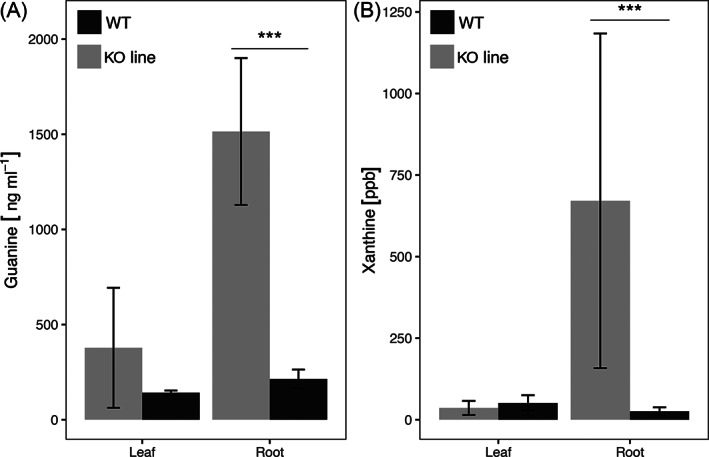
Guanine and xanthine content analysis in leaves and roots of the KO and WT plants. Barplots representing (A) guanine content, (B) xanthine content in KO (in grey), and WT (in black) in the leaves and roots sampled at 3 weeks (21 days) growth stage. In the roots of the KO and WT lines, there was a difference between the KO and WT plants in both the guanine and xanthine content (*t*(3) = 5.79, *P* = <0.001 and *t*(3) = 2.17, *P* = 0.06, respectively). No significant difference was noted in either the guanine or xanthine content in the leaves at the same growth stage

To prove GDA activity in vivo, an *OsGDA1* knockout (KO) line (TRIM M0039637; Hsing et al., 2007) of Tainung 67 (T67) was used. Immunodetection‐mediated analysis showed that the KO line did not contain the full‐length protein, while the wild type (WT) did (Figure S3). Due to the potential arrest of guanine conversion into xanthine in the KO line, more guanine and less xanthine was expected in the KO line compared to the WT plant as illustrated in the biosynthetic pathway modeled on information from Ashihara et al. (2018; Figure S4). Assessment of the guanine and xanthine content revealed the expected excess of guanine in the roots and leaves of the KO compared to the WT (Figure 2A). Although, the content of xanthine was expectedly less in the KO compared to the WT in the leaves (Figure 2B), it was, unexpectedly, higher in the KO roots compared to the WT. A combination of two reasons could be responsible for this observation. First, the lack of GDA activity may promote the conversion of guanosine to xanthine through xanthosine. The enzyme that converts guanosine to xanthosine is the guanosine deaminase, which is 28 times more active than the guanine deaminase in tea plants (Negeshi et al., 1994). Second, xanthine is preferentially synthesized in the roots just as in tea plants (Deng and Ashihara, 2015). The need for xanthine in roots most likely drives the guanosine to xanthine pathway through the more effective guanosine deaminase, causing more xanthine build up in the KO. Nevertheless, the results in Figures 1 and 2 support the in vitro and in vivo GDA activity, respectively, for the newly identified *OsGDA1*.

Genes for GDA are known from microbial, invertebrate, and animal systems (Fernandez et al., 2009). In plants GDA enzyme activity leading to xanthine, is known in tea leaf extracts (Negishi et al., 1994), but no plant gene is identified as *GDA*. In Arabidopsis, the plant‐specific guanosine deaminase (*AtGSDA*) is proposed to be the major source of xanthine through the xanthosine phosphorylase enzymatic reaction that catabolizes xanthosine to xanthine (Dahncke and Witte 2013). The putative rice ortholog of *AtGSDA*, LOC_Os03g61810 (Dahncke and Witte, 2013), is variously annotated as a nucleoside/nucleobase deaminase, with at least one computational prediction of it being a *GDA*. However, no experimental proof exists for its activity as a deaminase for any nucleoside/nucleobase. An alignment of the predicted protein sequence of LOC_Os03g61810 with *OsGDA1* revealed 22.16% identity and a lack of all the residues for the active site and ligand and substrate binding sites of GDA (Figure S5A). In fact, as predicted by Dahncke and Witte (2013), LOC_Os03g61810 may indeed be the rice guanosine deaminase because the active site residues of the AtGSDA (At5g28050) and it's human ortholog were conserved in the protein coded by the gene at LOC_Os03g61810 (Figure S5B). If the putative rice GSDA (*OsGSDA1*) takes up the compensatory role for xanthine synthesis through xanthosine, it should be upregulated in the KO plant compared to the WT plant. Figure S6 shows a trend for upregulation of the putative *OsGSDA1* transcript of LOC_Os03g61810 in the KO plant albeit with a large variation (large error bar for KO in Figure S6). One reason for the large variation could be the accumulation of guanine in the KO line plants and the possible resulting variation in the extent of its conversion to guanosine (Figure S4). Although preliminary, this result suggested LOC_Os03g61810 to be potentially responsive to the lack of OsGDA1 in the KO plant, and also, it's in silico similarity to AtGSDA suggested that if functional as an *OsGSDA1*, it could convert guanosine to xanthosine (Figure S5B). The contribution of the alternate routes of adenosine‐to‐xanthosine or guanosine‐to‐xanthosine conversion (Figure S4) may be different in different biological replicates, which might explain the high variation in transcript abundance in the replicates (Figure S6).

We could not test for the potential 2′‐deoxyadenosine deaminase activity of OsGDA1 in vivo due to technical limitations. However, the metabolic pathway (Figure S4) suggests that the KO of the gene performing such an activity might reduce the content of 2′‐deoxyinosine. The 2′‐deoxyinosine to xanthosine synthesis through Inosine Monophosphate (IMP) is known (Fox and Kelly, 1978). Thus, in the KO line, the pathway might be pulled to use more adenosine to make IMP. This would limit the conversion of adenosine to 2′‐deoxyadenosine. Such a pull could be useful since adenosine might already be in excess due to the inactivity of OsGDA1. Additionally, the substrate demand for adenosine to make more IMP will tip the balance favoring the production of xanthosine and xanthine, compensating for the lack of guanine conversion to xanthine in the KO. These are logical assumptions because cases of deaminases differentiating between closely related nucleosides such as adenosine and 2′‐deoxyadenosine are known (Miller and Maier, 2013) but the existence and utilization of 2′‐deoxyadenosine for deamination remains to be shown. No gene or gene product for 2′‐deoxyadenosine deaminase is characterized in plants to the best of our knowledge.

In summary, we demonstrated that in rice plants LOC_Os12g28270 was the first plant GDA gene to be identified (*OsGDA1*). Its product deaminated guanine to xanthine in vitro, as known in other systems, and most likely in vivo as well, as suggested mainly by the increased guanine in the KO plants. We also provide preliminary results to suggest that LOC_Os03g61810 might be the *OsGSDA1*, and finally that an in vivo function of OsGDA1 in deaminating 2′‐deoxyadenosine to 2′‐deoxyinosine, as it does in vitro, could support our results that the expected reduction in xanthine content in the KO leaves is not extensive while the KO roots actually show more xanthine.

### 3.2 Insights on OsGDA1 from mutant distribution in wild and cultivated rice

A number of nonsynonymous substitutions in *OsGDA1* between Vandana and Way Rarem (Figure S7) prompted a deeper consideration of variants of this gene in a broader pool of genotypes. Analysis of the 3 K genome sequence data (Alexandrov et al., 2015; The 3000 Rice Genome Project, 2014) revealed a high degree of variation in this gene. Based on 11 polymorphic sites (highlighted across the columns in pink and gray in Table S2), 70% of genotypes carried one or more SNPs that differ from the Nipponbare reference genome. Various classes of SNPs, such as synonymous, missense, stop, splice variant, etc. were distributed throughout the gene in its 3′ and 5′ UTRs, introns and exons. To ascertain the importance of such variation in *OsGDA1*, accessions with more than 15x genome coverage were selected. These were a mix of the cultivated *O. sativa* sub‐populations and wild species, and were used for nucleotide diversity analysis. All positions containing gaps and missing data were excluded. There were a total of 131 polymorphic nucleotide sites in the final dataset. Results revealed that there was little to no decrease in nucleotide diversity in cultivated rice compared to the wild rice (Table S3). This suggested *OsGDA1* may not have been a target of artificial selection during rice domestication. In the aromatic rice subgroup the diversity was marginally higher than in wild rice. The very low sample size (*n* = 5) most likely skewed the diversity measures for this group.

**FIGURE 3:**
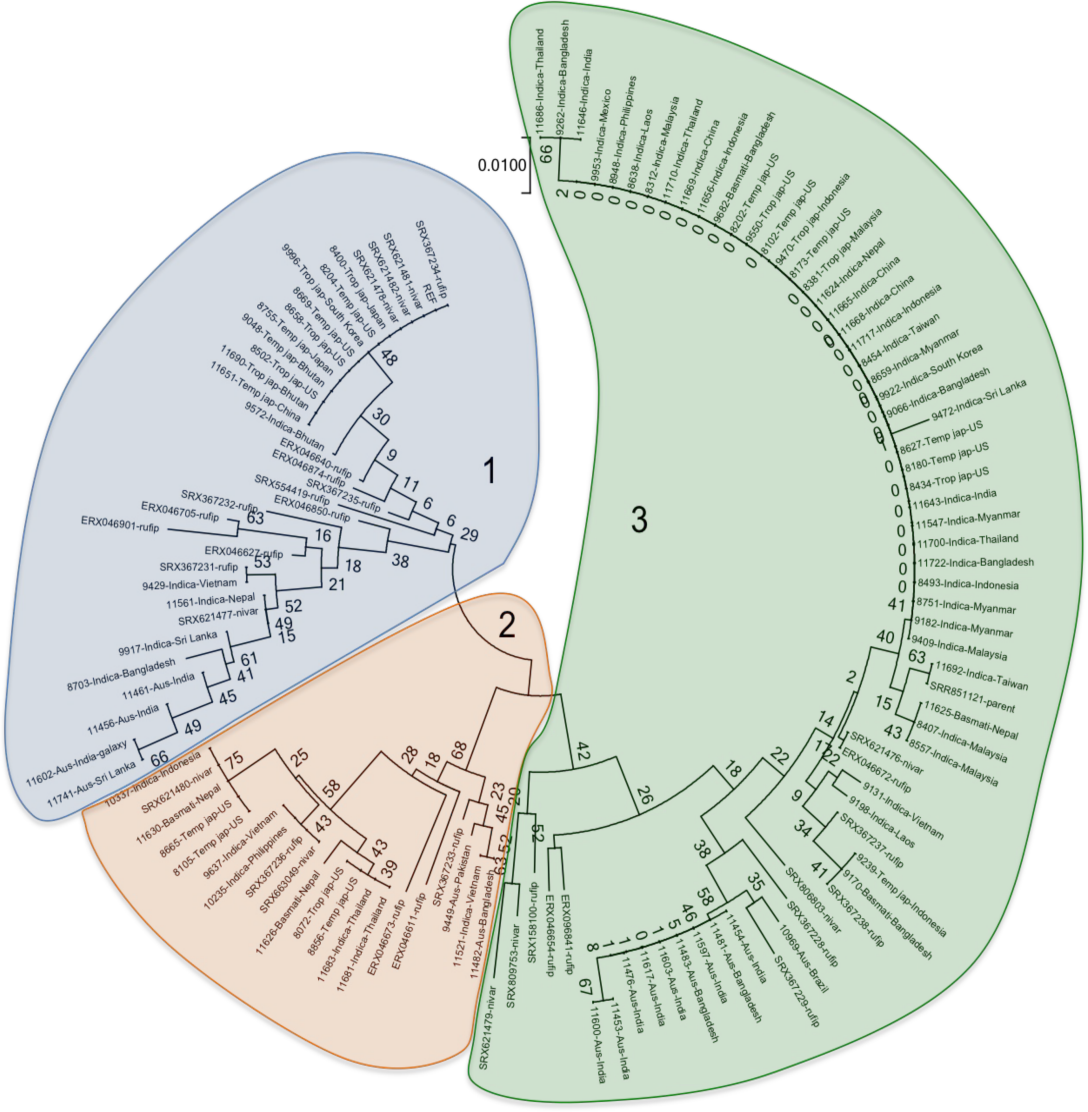
Neighbor‐joining tree of wild and cultivated rice based on the protein sequence. The percentage of replicate trees in which the associated accessions clustered together in the bootstrap test (1000 replicates) are shown next to the branches. The tree is drawn to scale, with branch lengths in the same units as those of the evolutionary distances used to infer the phylogenetic tree. Evolutionary distances were computed using the Poisson correction method and are in units of the number of amino acid substitutions per site. The analysis involved 472 amino acid sequences. All positions containing gaps and missing data were excluded. There were a total of 131 positions in the final dataset. Evolutionary analyses were conducted in MEGA7. The three clades are shown with numbers 1, 2, and 3

The results on differential protein activity of OsGDA1 in Vandana and Way Rarem (Figure 1; Figure S2) prompted a protein phylogeny analysis for potential functional differences. Three weakly diverged groups were identified (Figure 3; Clades 1, 2, and 3), each of which included both cultivated and wild accessions. This suggested evolutionary divergence at the *OsGDA1* locus that predates the domestication process.

Effects of amino acid mutation on protein activity can be predicted by the associated protein destabilization score through changes in Gibbs free energy (ΔΔG or DDG: change in Gibbs free energy; Folkman et al., 2016). When six random protein sequences from each Clade were analyzed for protein destabilizing mutations (DM), Clade 1 contained two proteins with one DM each, and Clade 2 contained four proteins with one DM each, whereas in Clade 3 all six proteins contained DM, two each in three proteins and a single one in the other three proteins (Table S4). Sequences in Clade 1 were similar to the Nipponbare reference genome and the mutations lacked a discernible pattern. The gene/protein sequence of T67, the WT accession, which was used in KO studies, is identical to Nipponbare. Clade 2 was defined by DM that included M300L, A304S and R454S. Clade 2 proteins were less similar to Nipponbare than those in Clade 1. Quantitatively and qualitatively most DMs, i.e. E450G, T109M, I452R and R454S occurred in Clade 3, most dissimilar to Nipponbare, and in many cases one protein contained more than one highly destabilizing mutations. Mutations in wild species *Oryza rufipogon* in Clade 3 were similar in the cultivated *O. sativa* temperate *japonica* accession from the US and an *Aus* rice sub‐type accession from India. Also, in Clades 1 and 2, the mutations of wild species *O. rufipogon* and *O. nivara* could be found in cultivated *O. sativa*.

Comparison of Table S4 and Figure S7 suggested that Vandana and Way Rarem both belonged to Clade 3 but their protein sequences differed at multiple sites (Figure S7). This included changes in amino acids that predicted critical alterations in protein post‐translational modifications such as phosphorylation and amidylation. Importantly, there was the L279M mutation in the substrate binding site. The L to M mutation is known to decrease protein stability (Lipscomb et al., 1998). Other residues of the ligand binding, substrate binding and active site were identical in the two proteins. Yet, the in vitro activity of the Vandana protein was reduced towards both, guanine and 2′‐deoxyadenosine substrates (Figure S2).

These results reiterated the evolutionary divergence before domestication, but also suggested that during the evolutionary process the accessions within each Clade accumulated variations in the functionally critical residues which may have been artificially selected. Since most amino acid changes were destabilizing mutations, there is a possibility of multiple selection events for null alleles suggesting a value for negative regulation of a trait or traits by *OsGDA1* during evolution/breeding. Our previous results suggest that *OsGDA1* may most likely have a role in the negative regulation of root architecture, especially in the proportion of small lateral roots (Dixit et al., 2015). More root biomass is generally associated with stress tolerance. Rice *semidwarf 1* (*sd1*), a gibberellin oxidase gene, is a good example of selection for a null allele in domesticated rice. Regardless of genetic background, the *Sd1* null allele negatively regulates plant growth, a useful trait underpinning the Green Revolution rice cultivars (Zhang et al., 2020).

### 3.3 Effect of OsGDA1 KO on the metabolites of the SAM to xanthosine pathway

**FIGURE 4:**
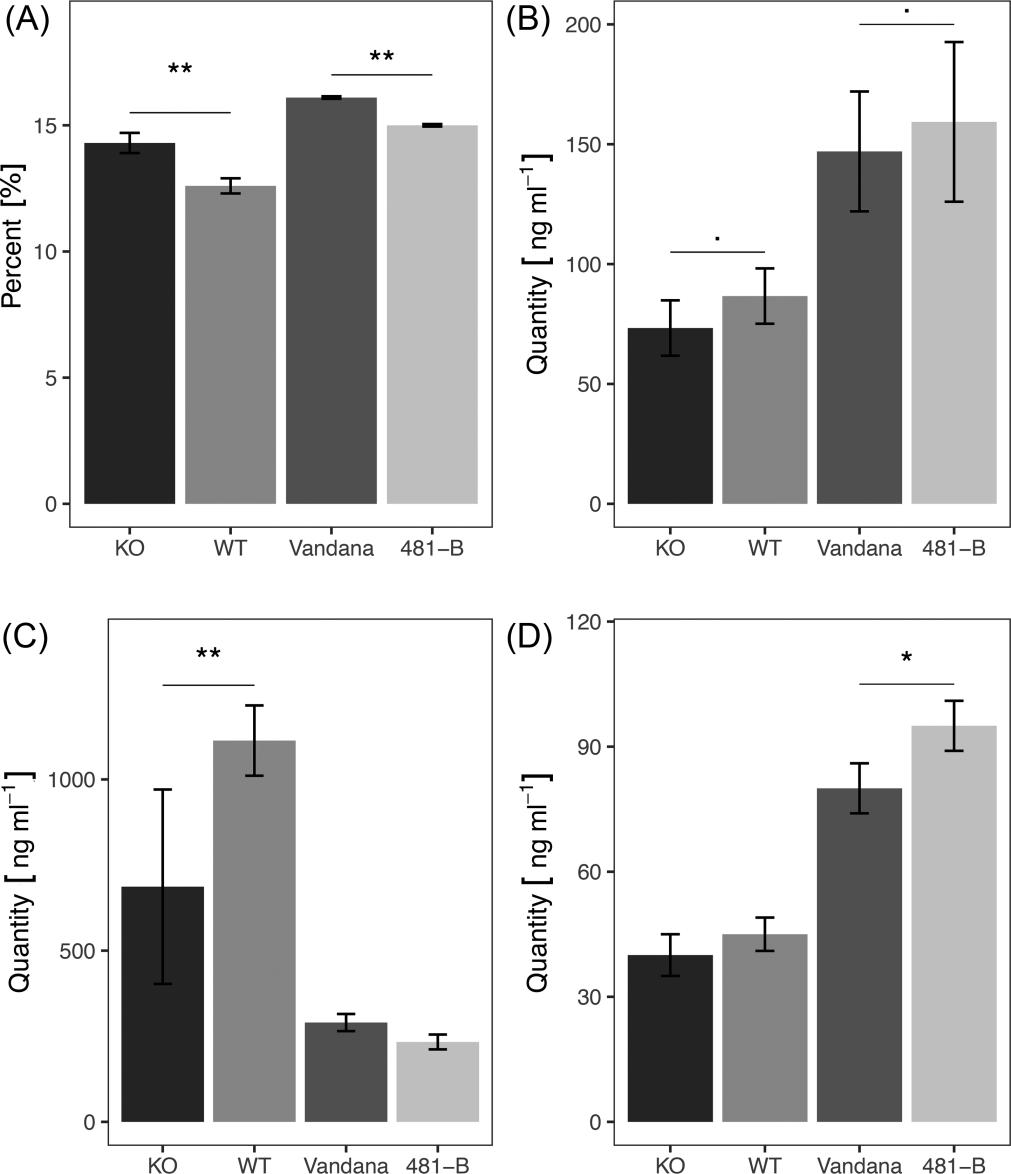
Cytosine methylation, SAH, SAM, and adenosine content analysis in two contrasting sets of rice lines. Barplots representing (A) percentage of genomic cytosine methylation, (B) S‐Adenosyl homocysteine content, (C) S‐Adenosyl methionine content, and (D) adenosine content in KO, WT, Vandana, and 481‐B in leaves sampled at 21 day growth stage. Significance levels between KO and WT indicate *P* < 0.01 represented with “**”*P* < 0.05 with “*” and *P* < 0.1 with “.”. There was a difference between KO and WT lines in the percentage of methylation (*t*(5) = 4.65, *P* = 0.002); in the content of SAM [t (5) = −3.16, *P* = 0.024); and also in SAH content (*t*(5) = −1.85, *P* = 0.10) but not in the adenosine content. Differences were also found between Vandana and 481‐B in the percentage of methylation (*t*(3) = 7.82, *P* = 0.008), SAH content (*t*(3) = −2.56, *P* = 0.081) and adenosine content (*t*(3) = −2.97, *P* = 0.04) but not in SAM content

If as suggested by Ashihara et al. (2013) for tea plants, the main route of xanthine synthesis is through the SAM‐SAH‐adenosine pathway, then there would be limitations on xanthine synthesis through guanine in the KO line. This could place an increased demand on SAM, SAH and adenosine to synthesize xanthine through guanosine (Figure S4). Hence, compared to the WT the SAM, SAH and adenosine content could be less in the KO than in the WT. This was borne out through targeted metabolite analysis (Figure 4B–D). The Figure 4 also shows the trends for the comparative content of these metabolites in Vandana and 481‐B, which is the near‐isogenic line (NIL) that contains the Way Rarem q*DTY*_*12.1*_ gene‐cluster introgressed to Vandana (Dixit et al., 2015). Thus, Vandana could be analogous to the KO and 481‐B, a reconstituted WT. Results shown in Figure 4 support the analogy in that SAH and adenosine contents are less in Vandana than in 481‐B (Figure 4B,D), while the SAM content is similar in the two lines (Figure 4C).

The NIL 481‐B is generated by introgression of a gene‐cluster containing the *OsGDA1* rather than just the single functional *OsGDA1*. Our attempt at estimating the guanine and xanthine content in Vandana and 481‐B was successful in Vandana for guanine and xanthine but only for guanine in 481‐B. Missing the data for xanthine content in 481‐B was a limitation. However, a trans‐effect of *OsGDA1* through xanthine could be used as a measure of correspondence between the KO/WT and the Van/481‐B lines. For example, *OsGDA1* KO line has less xanthine in the leaves compared to the WT but more of it in the roots of the KO than in the WT (Figure 2B). If the same happened in Vandana and 481‐B, then the effect of xanthine content could be compared indirectly in the leaves and roots through how it might affect any associated metabolites/processes. Fortunately, a previous transcriptome analysis between Vandana and 481‐B was available (Dixit et al., 2015). In the 10 frequent GO slim terms for the upregulated set of genes, 481‐B in comparison to Vandana, had a higher percentage of genes upregulated for all GO terms except for the “nucleobase, nucleoside, nucleotide and nucleic acid metabolism.” As a corollary, this was the category in which the percentage of genes upregulated in Vandana was maximum (Table 1).

**TABLE 1:** Top 10 GO terms for genes upregulated in 481‐B

	GO slim term	% Genes upregulated in Vandana*	% Genes upregulated in 481‐B*
GO:0006950	Response to stress	20.72	19.54
GO:0006139	Nucleobase, nucleoside, nucleotide and nucleic acid metabolic process	*20.72*	14.11
GO:0006464	Protein modification process	12.72	14.017
GO:0006810	Transport	10.90	14.51
GO:0009628	Response to abiotic stimulus	10.54	12.33
GO:0009607	Response to biotic stimulus	7.27	6.12
GO:0007165	Signal transduction	6.18	8.29
GO:0009056	Catabolic process	6.18	6.41
GO:0009908	Flower development	3.27	2.66
GO:0009790	Embryo development	1.45	2.27

* The GO terms that were upregulated supported the role of the gene cluster in stress tolerance.

Among the nucleobase/nucleoside/nucleotide metabolism genes, four rice nucleobase‐ascorbate transporters (NAT; LOC_Os01g55500; LOC_Os08g28170; LOC_Os07g30810; LOC_Os09g15170) are particularly similar to the xanthine/allantoin/uric acid transporters/permeases characterized from Arabidopsis (Desimone et al., 2002). For the *OsGDA1*, KO line more xanthine was noted in the roots but less in the leaves (Figure 2B). This difference could manifest in the upregulation of the NATs in the roots and down‐regulation in the leaves of the KO plants compared to the roots and leaves of the WT, respectively. If Vandana and 481‐B were analogous to KO and WT, similar results could be expected.

Two rice NATs, (LOC_Os01g55500 and LOC_Os07g30810), were more similar to the Arabidopsis xanthine/uracil permeases (At2G34190 and At4G380590, respectively). The NAT at LOC_Os09g15170 was 83% similar to At2G34190. Hence, these three NATs were analyzed by QRT‐PCR in leaves and roots. The results did bear out the hypothesis by a trend of upregulation of the NATs in the roots of the KO where more xanthine was present compared to the WT, but also in Vandana compared to 481‐B (Table S5). The expression of NATs in the leaves was almost similar between the KO and WT, and Vandana and 481‐B. These results supported the correspondence between Vandana versus 481‐B and the KO versus WT for the effect of *OsGDA1* on the SAH‐to‐xanthine pathway.

### 3.4 The SAH to xanthine pathway perturbations affect the epigenome

Results presented in the previous section revealed that a difference in the amount of SAH could potentially occur due to a difference in the functionality of OsGDA1. The KO line with a nonfunctional OsGDA1 when compared to the WT for SAM, SAH and adenosine content provided indications for increased utilization of these metabolites in the KO line (Figure 4B–D).

If *OsGDA1* functions also for deaminating 2′‐deoxyadenosine in vivo, and thus, the KO line is also deficient in that reaction, the SAH‐to‐xanthosine pathway would experience a further pull in the forward direction. The lack of 2′‐deoxyinosine, which feeds the pathway towards xanthosine, will add to that pull. Additionally, 2′‐deoxyadenosine is an inhibitor of SAHH (Abeles et al., 1982) hence its accumulation in the KO may lead to accumulation of SAH, which may be diverted to adenosine, which in turn may feed towards xanthosine instead of being converted to its already accumulating deoxy‐moiety. This reiteratively explains the decreased SAH and adenosine. However, as mentioned earlier, the 2′‐deoxyadenosine and its genetic controls are yet unknown in rice.

The demonstrated changes in the cellular SAH content are a critical modification. SAH is a potent inhibitor of the DNA methyltransferases (DMTs; James et al., 2002). Rocha et al., (2005) and Mull et al., (2006) demonstrated that increased SAH content due to the silencing/mutation of S‐adenosyl homocysteine hydrolase (SAHH) in Arabidopsis causes reduced DNA cytosine methylation. On the contrary, reduction in SAH content might increase genomic methylation. If this was true in rice then the KO lines, due to reduced SAH content, should have more genomic DNA methylation compared to the WT. Moreover, due to the correspondence established between the KO and Vandana, the latter should also have more genomic methylation than 481‐B. We confirmed this hypothesis through multiple evidence.

Comparing the *OsGDA1* KO line to the WT plant for genomic methylation, preliminary analysis using methylation sensitive isoschizomers *MspI* and *HpaII* revealed hypermethylated genomic DNA in the KO plant (Figure S8). Three additional independent lines of evidence reiterated that the genome level cytosine methylation was more in the KO than in the WT plants. The number of cytosine methylation sites detected through the methyl‐sensitive amplification polymorphism (MSAP), once again using the methylation sensitive *Msp*I/*Hpa*II isoschizomers, were more in the KO plant (Figure S9A). Similarly, global DNA methylation detected through anti‐5‐mC antibody was more in the KO plant (Figure S9B). For a final confirmation HPLC‐mediated quantitative detection of percent methylation of the genomic DNA was used, and it was greater in the KO compared to the WT and in Vandana compared to 481‐B (Figure 4A), thus confirming similar effect of OsGDA1 in different genetic backgrounds. Taken together these results suggested that increased genomic methylation in the KO and Vandana was most likely related to a decrease in SAH content, which in turn was possibly due to the upregulation of the SAH‐to‐xanthosine pathway due to the KO or sub‐optimal *OsGDA1*.

Unlike the similar evidence from a previous study which manipulated *SAHH*, which acts directly on SAH, *OsGDA1* is a downstream enzyme. Thus, the results suggested a tightly regulated, largely linear pathway, leading to xanthine. This pathway is suggested as the most active route to xanthine synthesis in tea and coffee (Ashihara et al., 2011), where xanthine is a precursor of theine and caffeine synthesis, respectively. In mammalian neuronal cells the fate of guanine is largely deamination into xanthine (Brosh et al., 1992). In plants, maintaining the cellular pool of xanthine and its metabolism is important to regulate the level of ureides and allantoate for optimal plant growth through nutrient storage and remobilization (Brychkova et al., 2008; Hesberg et al., 2004). Hence compensation for decrease of xanthine through guanosine to xanthosine, via a pull on the upstream metabolites in the KO plants can be expected.

The effect of *OsGDA1* at the genome level by affecting DNA methylation, and at the metabolome level by affecting highly networked metabolites (SAM, SAH, adenosine), make it an important pleiotropic gene. Additionally, the basic processes of DNA and protein synthesis, sub‐cellular transport, signaling, cellular energetics, and more, involve guanine nucleotides. The GTP‐binding proteins (G‐Proteins) are active in many cellular processes which affect important agronomic traits such as response to abiotic and biotic stress, symbiosis, seed yield, nitrogen use efficiency, and organ size (Pandey, 2019). Similarly, xanthine is involved in purine metabolism, ureides biosynthesis and nitrogen fixation. Silencing of xanthine dehydrogenase (XDH) in Arabidopsis leads to an over accumulation of xanthine causing growth retardation, abnormal fruit development and seed fertility, and leaf senescence (Nakagawa et al., 2007). In another study both suppression and overexpression of XDH was studied and the effects were expectedly opposite in the response to drought stress, the overexpression lines being more tolerant by maintaining photosynthesis and regulating the reactive oxygen metabolism (Han et al., 2018). Thus, *OsGDA1* gene identification is an important step in following up the effect on the affected processes and traits. Allele selection for *OsGDA1* in breeding programs, especially those involving genomic selection can be an important contributor due to its major pleiotropic effects.

In summary, we demonstrated that the activity of *OsGDA1* links upstream to the cellular SAH pool and epigenome modulation. Such a connection may be important to be explored in cases of GDA‐mediated human diseases (Kumar et al., 1979; Fernandez et al., 2010) where studies on the importance of guanine and xanthine on health are far more evolved.

## 4 CONCLUSIONS

In summary, our results suggested that the rice amidohydrolase at LOC_Os12g28270 encodes a guanine deaminase (*OsGDA1*) in plants. The OsGDA1 activity generates the expected product xanthine in vitro and most likely in vivo as well. Extensive variation exists in the *OsGDA1* gene, and it may have undergone repeated selection in rice for variation in functionally critical residues, most likely for null alleles. Selection for the lack of a functional allele may be useful for certain negatively controlled agronomic traits such as the root architecture. Lack of a functional *OsGDA1* allele may be compensated in rice by the as yet uncharacterized guanosine deaminase (*OsGSDA1*, likely LOC_Os03g61810, which is not yet annotated as an amidohydrolase) for xanthine homeostasis as in Arabidopsis. When the *OsGDA1* is knocked out or down regulated, it exerts a pull on the purine metabolism pathway for xanthine homeostasis, leading to a decrease in SAH content. The decrease in the SAH content can result in increased genomic methylation, in turn leading to gene silencing or expression. These results posit *OsGDA1* as an important pleiotropic gene for further studies for both upstream understanding of its effect on metabolic networks and epigenetics and for downstream translational value in breeding programs.

## AUTHOR CONTRIBUTIONS

Tamara Iglesias, Elisa Blanco Gonzalez, and Maria Montes Bayon participated in the experiments related to measuring the metabolites. Lin‐Feng Li and Kenneth M. Olsen conducted the phylogeny and evolutionary assessments. Naoki Yamamoto analyzed the transcriptome data. Dhananjay Gotarkar, Toshisangba Longkumer, Amrit Kaur Nanda, and Berta Miro conducted the remaining experiments, analyzed the data and wrote the first draft. Berta Miro finalized the Figures, Tables, and formatting. Yue‐Ie Caroline Hsing provided critical plant material and information on the same. Ajay Kohli designed the project and the experiments, analyzed the data and finalized the manuscript. All authors reviewed and refined the manuscript.

## Supporting information

**FIGURE S1** Purified recombinant OsGDA1
**FIGURE S2** Comparison of enzyme activity between the Vandana and Way Rarem recombinant amidohydrolase
**FIGURE S3** Immunodetection of OsGDA1
**FIGURE S4** SAM‐to‐xanthosine pathway
**FIGURE S5** Protein sequence alignments for the rice GDA (LOC_OS12g28270) and the putative GSDA (LOC_Os03g61810)
**FIGURE S6** QRT‐PCR‐mediated expression of LOC_Os03g61810
**FIGURE S7** Alignment of the OsGDA1 protein sequences
**FIGURE S8** Genomic methylation analysis
**FIGURE S9** DNA cytosine methylation status
**TABLE S1** Nine chemicals tested for deamination by the putative OsGDA1
**TABLE S2** Missense (non‐synonymous) substitutions due to the SNPs in the OsGDA1 coding region
**TABLE S3** Nucleotide diversity (π) and genetic diversity (Watterson's Theta θW) of OsGDA1 in wild and cultivated rice accessions
**TABLE S4** Protein destabilization due to amino acid changes
**TABLE S5** qRT‐PCR results for three rice NATs
**TABLE S6** List of primers used for cloning and QRT‐PCR

## Data Availability

The data that support the findings of this study are available from the corresponding author upon reasonable request.
