# Supplementary material for: A drought‐responsive rice amidohydrolase is the elusive plant guanine deaminase with the potential to modulate the epigenome
Source: Physiol Plant. 2021 Apr 1;172(4):1853–66. doi: 10.1111/ppl.13392 (PMC8360030; doi:10.1111/ppl.13392)
Supplement: Supplementary file 1 — FIGURE S1 Purified recombinant OsGDA1 FIGURE S2 Comparison of enzyme activity between the Vandana and Way Rarem recombinant amidohydrolase FIGURE S3 Immunodetection of OsGDA1 FIGURE S4 SAM‐to‐xanthosine pathway FIGURE S5 Protein sequence alignments for the rice GDA (LOC_OS12g28270) and the putative GSDA (LOC_Os03g61810) FIGURE S6 QRT‐PCR‐mediated expression of LOC_Os03g61810 FIGURE S7 Alignment of the OsGDA1 protein sequences FIGURE S8 Genomic methylation analysis FIGURE S9 DNA cytosine methylation status TABLE S1 Nine chemicals tested for deamination by the putative OsGDA1 TABLE S2 Missense (non‐synonymous) substitutions due to the SNPs in the OsGDA1 coding region TABLE S3 Nucleotide diversity (π) and genetic diversity (Watterson's Theta θW) of OsGDA1 in wild and cultivated rice accessions TABLE S4 Protein destabilization due to amino acid changes TABLE S5 qRT‐PCR results for three rice NATs TABLE S6 List of primers used for cloning and QRT‐PCR [file PPL-172-1853-s001.pdf]

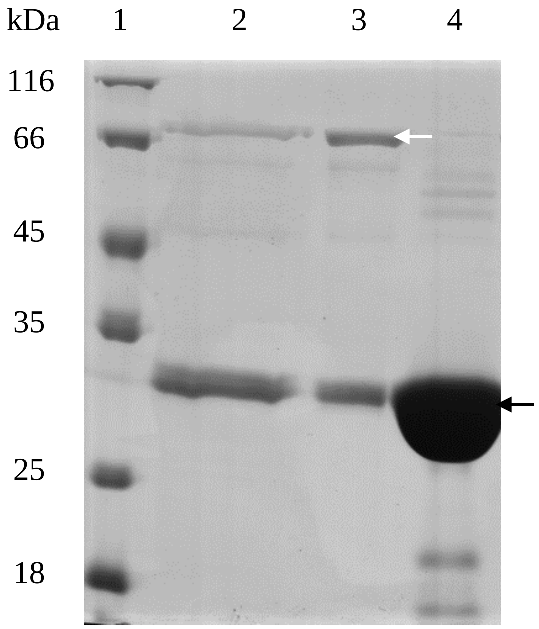

Supplementary Fig. 1. Purified recombinant OsGDA1. Lane 1 represents protein molecular weight marker, lanes 2 & 3 indicate GST-fused OsGDA1 (white arrow) of Way Rarem and Vandana respectively. Lane 4 shows the GST protein (black arrow). The loading of the lanes was unequal. For the enzyme assay, we quantified OsGDA1-GST band in comparison with the BSA standard band and used equal amount of the purified Vandana and Way Rarem protein.

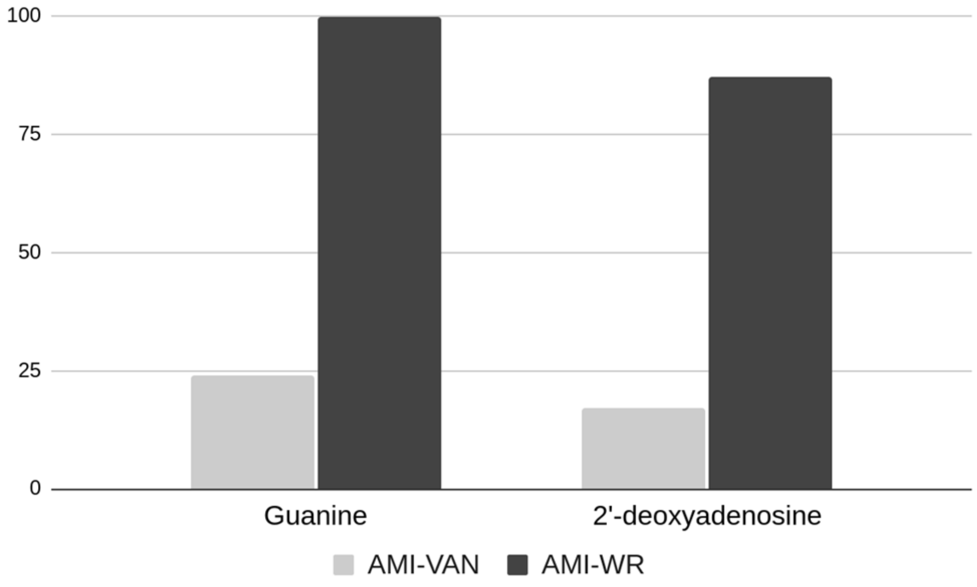

Supplementary Fig. 2. Comparison of enzyme activity between the Vandana and Way Rarem recombinant amidohydrolase (AMI) protein for deaminating guanine and 2’-deoxyadenosine. The rate of reaction is defined as the amount of substrate depleted per hour per ml reaction. The Vandana protein (grey bars) showed 76.48 and 46.30 pmoles/ml/min for guanine and 2’-deoxyadenosine respectively and the Way Rarem protein (black bars) showed 302.04 and 268.77 pmoles/ml/min against guanine and 2’-deoxyadenosine respectively. The activities are represented at 100% for the Way Rarem protein for guanine and other activities as the proportion of 100%.

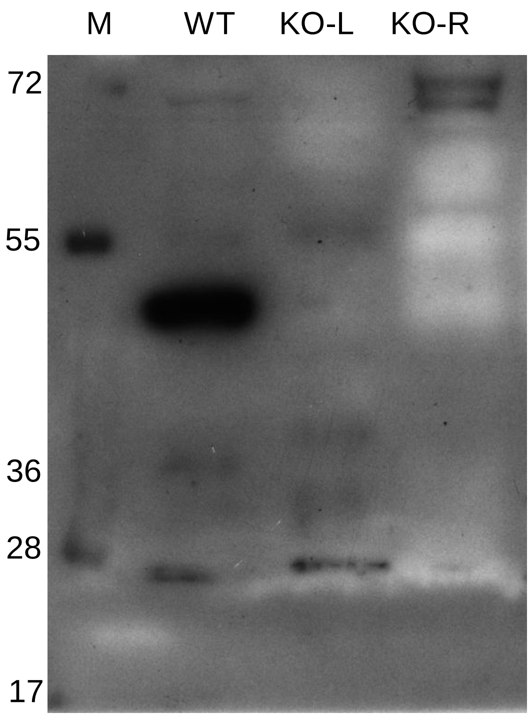

Supplementary Figure 3. Immunodetection of OsGDA1. M: molecular weight marker with kDa shown on the side in numbers. The protein was detected as a blob at ~50 kDa in the WT but not in the KO leaf (KO-L) or root (KO-R). Additional bands are non-specific bands due to the use of polyclonal antibodies.

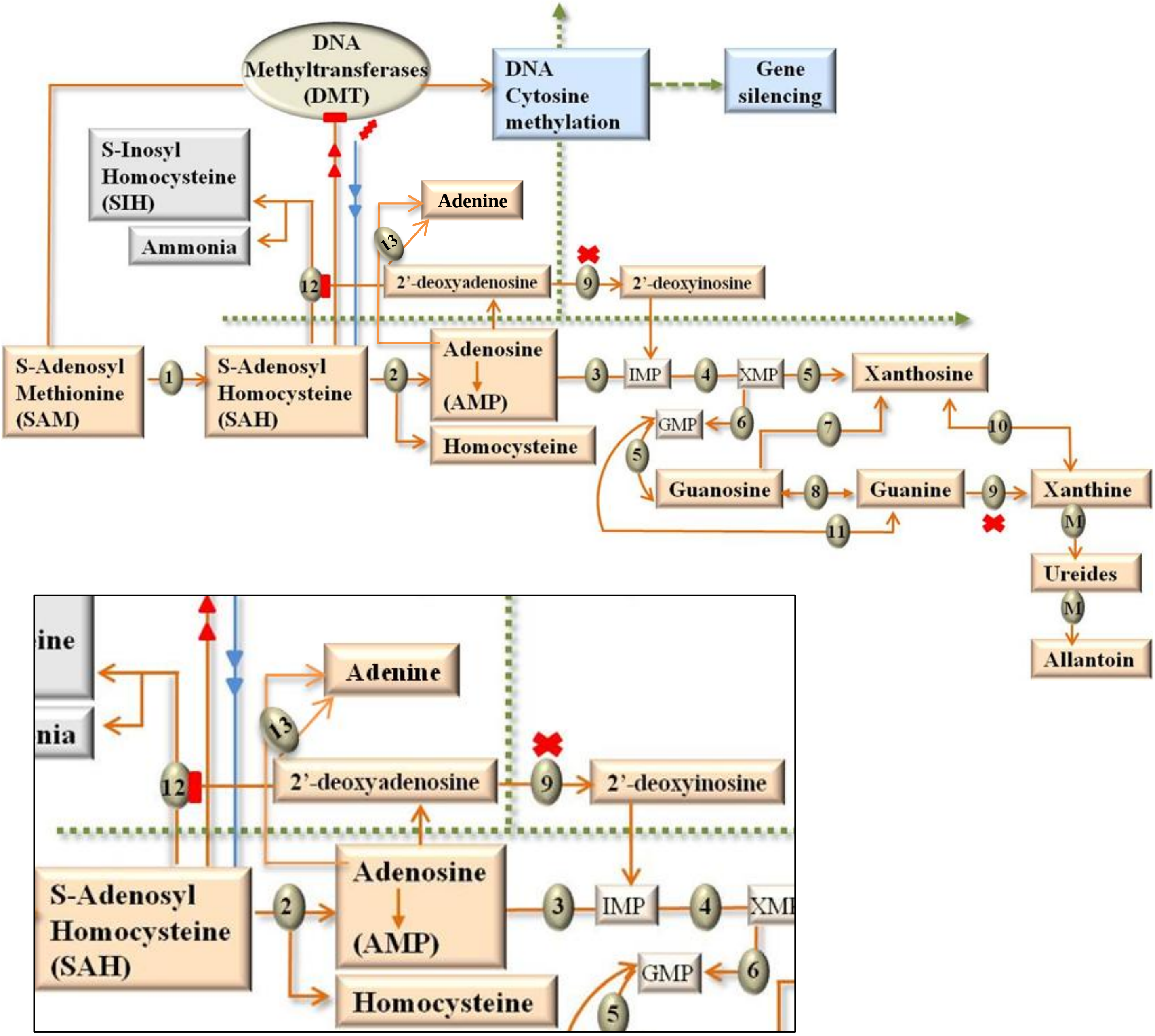

Supplementary Figure 4. SAM-to-xanthosine pathway. The SAM-to-xanthosine pathway in plants as modeled from Ashihara et al., 2018. Grey ellipses represent the pathway enzymes. 1 = AdoMet-dependent methyltransferase; 2 = SAH hydrolase; 3 = AMP deaminase; 4 = IMP dehydrogenase; 5 = 5' nucleotidase; 6 = GMP synthase; 7 = guanosine deaminase. 8 = guanosine phosphorylase; 9 = guanine deaminase; 10 = xanthosine phosphorylase; 11 = hypoxanthin guanine phosphoribosyltransferase; 12 = SAH deaminase; 13 = Adenosine nucleosidase. M = multiple steps. Increase in SAH, represented by the red arrows. Potent inhibition of DMTs by SAH and of SAH deaminase by 2'-deoxyadenosine, represented by the small red rectangle. Dotted green lines represent reactions 2 to 5 pushed in the direction of the green arrow when *OsGDA1* is dysfunctional, as represented by the red cross. This reduces the SAH content, (blue line and arrows), leading to release of DMT inhibition, and thus to increased DNA methylation and gene silencing, also represented by the dotted green lines. The blow out represents adenine metabolism as affected by *OsGDA1* KO.

A

```
LOC_Os12g28270.2      MAAAAAVADEVTVLHGGVVVSMDGGFRVFQDGAVAVAGDRIAAVGPSADVLSSFPGAAAT
LOC_Os03g61810.1      ------------MEEAQVVESKDGT----------------------ISVASAFAGHQEA
                                  : .. ** * **                        .* *:* *   :

LOC_Os12g28270.2      VDLAGRILLPGFVNTHVHTSQQLARGIADDVDLMAWLHGRIWPYESHMTEEDSYASTLLC
LOC_Os03g61810.1      VQDRDHK-----------------------------------------------------
                      *:   :

LOC_Os12g28270.2      GIELIRSGVTCFAEAGGQYVSEMARAVELLGLRACLTKSIMDCGDGLPPNWSSCSTDDCI
LOC_Os03g61810.1      ----------------------------FLSKAVEEAYQGVDCGHGGPFGAVVVRNDEIV
                                                  :*.  .  : . :***.* *       .*: :

LOC_Os12g28270.2      QSQKDLYEK-HHNTADGRIRIWFGLRQIMNATDRLLLETRDAAQKLNTGIHMHIAEIPYE
LOC_Os03g61810.1      VSCHNMVLDYTDPTAHAEV--------------------TAIREACKK------------
                       * :::  .  . **...:                    *:*.:*

LOC_Os12g28270.2      NELVMQTKGIDHGTVTYLEKIDFLRSNLLAAHSVWLNKPEIGHFLKADVKVSHCPASAMR
LOC_Os03g61810.1      -----------------LGKIELSDCEMY-------------------ASCEPCPMCF-G
                                       * **::  .::                    .. . ** .

LOC_Os12g28270.2      MLGFAPIREMLDSGVCVSLGTDGAPSNNRMSIVDEMYLACLINKGREAYITGTTNPTALP
LOC_Os03g61810.1      AVHLSRIKRLVY----------GAKA------------EAAIAIGFDDFIADALRGTAYY
                       : :: *:.::           ** :             . *  * : :*: : . **

LOC_Os12g28270.2      AETVLKMATINGAKAVLWDDEIGSLEVGKKADMVVVNPLIWSMVPVHDCIANIVYCMRTE
LOC_Os03g61810.1      QKANLEIRRADGNGALIAEQVFENT--KEK----------FRMY----------------
                       :: *::   :*  *:: :: : .    :*          : *

LOC_Os12g28270.2      NIESVMCNGRWIMREKKIVNLNEEEVIASAEKIARDLLARAGINLPNRMNYL
LOC_Os03g61810.1      ----------------------------------------------------
```

B

```
ADAT2_HUMAN           ------------------------MEAKAAPK---PAASGACSVSAEETEKWMEEAMHMA
At5g28050             MRKFLVNVYSLSITRSPECYISSELEAKDGTISVASAFSGHQQAVHDSDHKFLTQAVEEA
LOC_Os03g61810.1      ------------------MEEAQVVESKDGTISVASAFAGHQEAVQDRDHKFLSKAVEEA
                                              :*:* .      * :*  ..  :  .*:: :*:. *

ADAT2_HUMAN           KEAL-ENTEVPVGCLMVYNNEVVGKGRNEVNQTKNATRHAEMVAIDQVLDWCRQSGKSPS
At5g28050             YKGVDCGDGGPFGAVIVHNNEVVASCHNMVLKYTDPTAHAEVTAIRE---ACKKLNKI--
LOC_Os03g61810.1      YQGVDCGHGGPFGAVVVRNDEIVVSCHNMVLDYTDPTAHAEVTAIRE---ACKKLGKI--
                       :.:      *.*.::* *:*:* . :* * . .: * ***:.** :    *::  *

ADAT2_HUMAN           EVFEHTVLYVTVEPCIMCAAALRLMKIPLVVYGCQNERFGGCGSVLNIASADLPNTGRPF
At5g28050             -ELSECEIYASCEPCPMCFGAIHLSRLKRLVYGAKAEAAIAIGFDDFIADA-LRGTGVYQ
LOC_Os03g61810.1      -ELSDCEMYASCEPCPMCFGAVHLSRIKRLVYGAKAEAAIAIGFDDFIADA-LRGTAYYQ
                        :..  :*.: *** ** .*::* ::  :***.: *   . *    **.* *  *.

ADAT2_HUMAN           ------QCIPGYRAEEAVEMLKTFYKQENPNAPKSKVRKKECQKS
At5g28050             KSSLEIKKADGNGAAIA----EQVFQN-----TKEKFRLY-----
LOC_Os03g61810.1      KANLEIRRADGNGALIA----EQVFEN-----TKEKFRMY*----
                            :   *  *  *    : .:::      *.*.*
```

Supplementary Figure 5. Protein sequence alignments. (A) The rice GDA (LOC_OS12g28270) and the putative GSDA (LOC_Os03g61810) are aligned to show 22.16% similarity and lack of conserved residues (Green: ligand binding; Red: active site; Yellow: substrate binding) between the two sequences for GDA activity. **(B)** LOC_Os03g61810 had conserved residues with human (ADAT2_HUMAN) and Arabidopsis (At5g28050) adenosine/guanosine deaminase, suggesting that it was most likely the predicted rice GSDA.

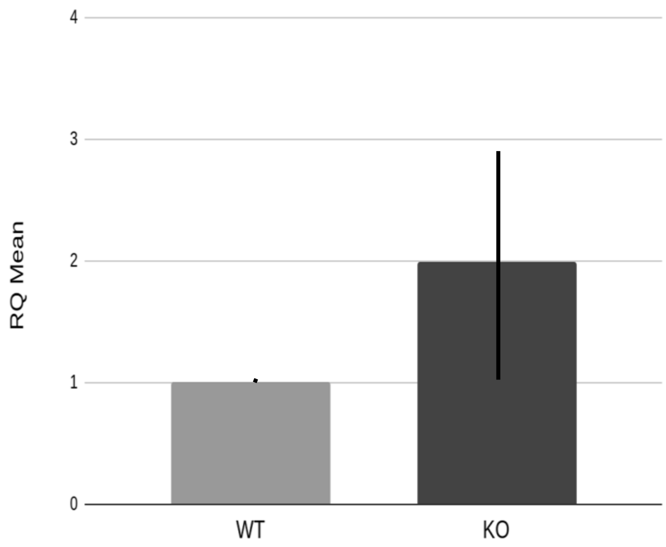

Supplementary Figure 6. QRT-PCR-mediated expression of LOC_Os03g61810. Transcript expression of the putative *OsGSDA1* was upregulated in the KO plant.

```
NB  MAAAAAVADEVTVLHGGVVVSMDGGFRVFQDGAVAVAGDRIAAVGPSADVLSSFPGAAAT 60
Van ---------------------MDGGFRVFRDGAVAVAGDRIAAVGPSADVLSSFPGAAAT 39
WR  ---------------------MDGGFRVFRDGAVAVAGDRIAAVGPSADVLSSFPGAAST 39
                         ********:****************************:*

NB  VDLAGRILLPGFVNTHVHTSQQLARGIADDVDLMAWLHGRIWPYESHMTEEDSYASTLLC 120
Van VDLAGRILFPGFVNTHVHTSQQLARGIADDVDLMTWLHGRIWPYESHMMEEDSYASTLLC 99
WR  VDLAGRILLPGFVNTHVHTSQQLARGIADDVDLMTWLHGRIWPYESHMTEEDSYASTLLC 99
    ********:*************************:************* ***********

NB  GIELIRSGVTCFAEAGGQYVSEMARAVELLGLRACLTKSIMDCGDGLPPNWSSCSTDDCI 180
Van GIELIRSGVTCFAEAGGQYVSEMARAVELLGLRACLTKSIMDCGDGLPPNWSSCSTDDCI 159
WR  GIELIRSGVTCFAEAGGQYVSEMARAVELLGLRACLTKSIMDCGDGLPPNWSSCSTDDCI 159
    ************************************************************

NB  QSQKDLYEKHHNTADGRIRIWFGLRQIMNATDRLLLETRDAAQKLNTGIHMHIAEIPYEN 240
Van QSQKDLYEKHHNTADGRIRIWFGLRQIMNATDRLLLETRDAAQKLNTGIHMHIAEIPYEN 219
WR  QSQKDLYEKHHNTADGRIRIWFGLRQIMNATDRLLLETRDAAQKLNTGIHMHIAEIPYEN 219
    ************************************************************

NB  ELVMQTKGIDHGTVTYLEKIDFLRSNLLAAHSVWLNKPEIGHFLKADVKVSHCPASAMRM 300
Van ELVMRTKGIDHGTVTYLEKIDFLRSNLLAAHSVWLNKPEIGHFLKADVKVSHCPASAMRM 279
WR  ELVMRTKGIDHGTVTYLEKIDFLRSNLLAAHSVWLNKPEIGHFLKADVKVSHCPASAMRL 279
    ****:******************************************************:

NB  LGFAPIREMLDSGVCVSLGTDGAPSNNRMSIVDEMYLACLINKGREAYITGTTNPTALPA 360
Van LGFAPIREMLDSGVCVSLGTDGAPSNNRMSIVDEMYLACLINKGREAYITGTTNPTALPA 339
WR  LGFSPVREMLDSGVCVSLGTDGAPSNNRMSIVDEMYLACLINKGREAYITGTTNPTALPA 339
    ***:*:******************************************************

NB  ETVLKMATINGAKAVLWDDEIGSLEVGKKADMVVVNPLIWSMVPVHDCIANIVYCMRTEN 420
Van ETVLKMATINGAKAVLWDNEIGSLEVGKKADMVVVNPLIWSMVPVHDCIANIVYCMRTEN 399
WR  ETVLKMATINGAKAVLWDNEIGSLEVGKKADMVVVNPLIWSMVPVHDCIANIVYCMRTEN 399
    ******************:*****************************************

NB  IESVMCNGRWIMREKKIVNLNEEEVIASAEKIARDLLARAGINLPNRMNYL 471
Van IESVMCNGRWIMREKKIVNLNEEEVIASAGKRASDLLARAGINLPNRMNYL 450
WR  IESVMCNGRWIMREKKIVNLNEEEVIASAEKIASDLLARAGINLPNRMNYL 450
    ***************************** * * *****************
```

Supplementary Figure 7. Alignment of the OsGDA1 protein sequences. The Vandana (VAN) and Way Rarem (WR) sequences differed at multiple sites including at a residue important for the substrate binding L249M. Green = ligand binding site; yellow substrate binding site; red = active site; purple = amino acid change sites between VAN and WR and Brown = amino acids that are different from the Nipponbare (NB) protein sequence used as the reference.

A

B

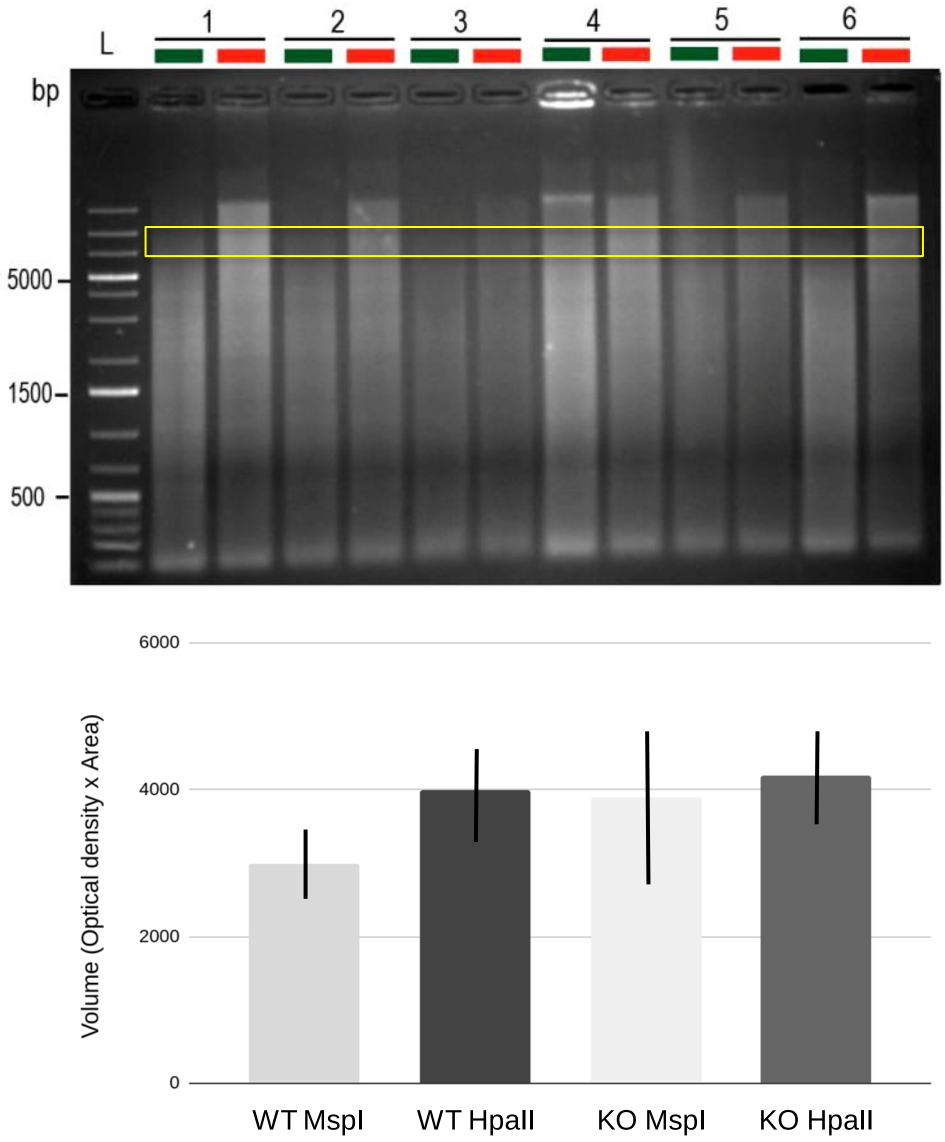

Supplementary Figure 8. Genomic methylation analysis. **(A)** Leaf genomic DNA samples were digested with methylation sensitive isoschizomers *Msp*I (green rectangle) and *Hpa*II (red rectangle). The recognition site CCGG is not cleaved by *Hpa*II if the internal C is Methylated. However, *Msp*I can cleave the DNA irrespective of the methylation status of the recognition site. Three independent biological replicates each of the WT DNA (samples 1, 2 and 3) and the KO DNA (samples 4, 5 and 6) were assayed. Apparently, the *Hpa*II digestion in samples 1, 2 and 3 was better than in samples 4, 5 and 6. The densitometry analysis within the yellow rectangle also supported this observation, whereby **(B)** the average intensity of the *Hpa*II digest lanes was more in the KO than in the WT plant indicating less digestion and hence hypermethylation of the genomic DNA in the KO plant.

A

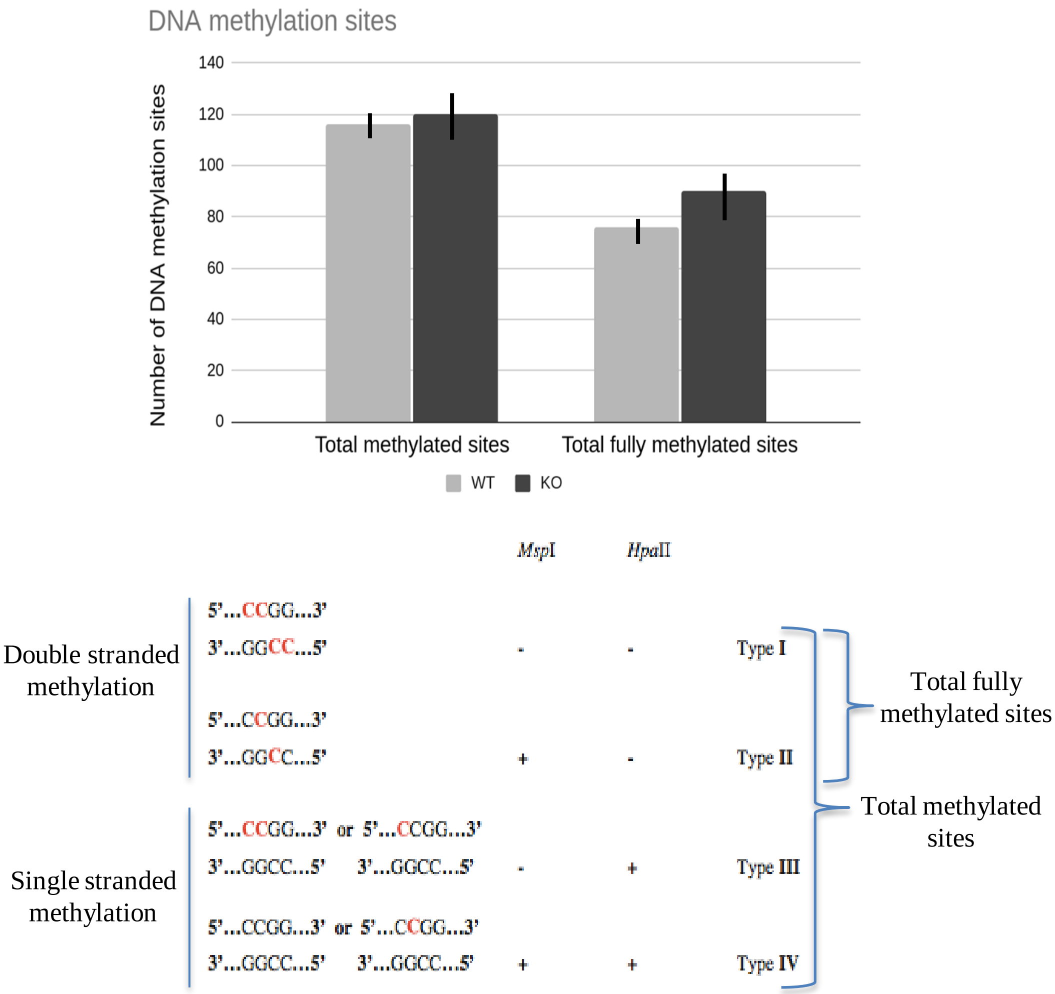

B

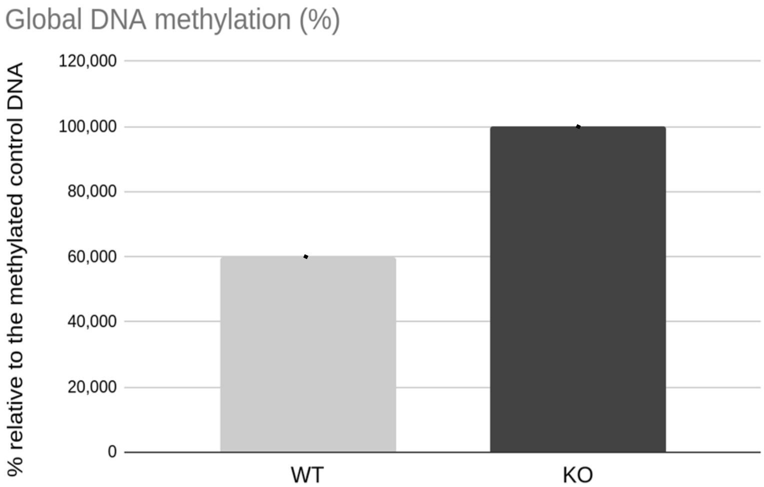

Supplementary Figure 9. DNA cytosine methylation status. Differential genomic methylation detected by **(A)** *Msp*I/*Hpa*II isoschizomers. Various possibilities of C methylation in the RE target site CCGG shown in red C, in the table below, lead to comparative presence (+) or absence (-) of bands with AFLP primers and the combination indicates 'Types' of methylation. The KO plant revealed more methylation. **(B)** The anti-5mC antibody-mediated estimation of genomic methylation also revealed more methylation in the KO plant.

Supplementary Table 1. Nine chemicals tested for deamination by the putative OsGDA1. Only guanine and 2'-Deoxyadenosine were deaminated.

S. No.	Substrate	Activity
1	Adenine	–
2	Adenosine	–
3	2'-Deoxyadenosine	+
4	5'-Deoxyadenosine	–
5	5'-Deoxy-5'-(methylthio)adenosine	–
6	Guanine	+
7	Guanosine	–
8	S-adenosyl homocysteine	–
9	Atrazine	–

Supplementary Table 2. Missense (non-synonymous) substitutions due to the SNPs in the *OsGDA1* coding region along the 10 exons of LOC_Os12g28270. The first column is the SNP position in bp, the second column indicates specific location in the gene. The 'Change' column indicates the amino acid change (from/to). Due to possible sequencing errors, SNPs showing more than 10 to 12% heterozygosity are not illustrated. The total number of missense mutations was 26, including one position (purple) with two possible mutations. The bp location in blue differentiate between the *indica* and *japonica* subpopulations. Positions highlighted in beige and grey are the 8 and 3 positions where ~70% or ~50% of the varieties respectively contain the SNP in relation to the reference genome of Nipponbare.

			Number of varieties		
SNP bp	Location in gene	Change	Homozygous	Heterozygous	% Heterozygosity
16712726	Exon 1	A/V	64	7	10.9
16712726	Exon 1	A/-	253	0	0.0
16712725	Exon 1	A/-	253	3	1.2
16712691	Exon 1	V/I	20	2	10.0
16712689	Exon 1	R/C	210	15	7.1
16712681	Exon 1	S/T	2301	31	1.3
16712680	Exon 1	S/R	2303	32	1.4
16712654	Exon 1	Q/R	2291	97	4.2
16712653	Exon 1	Q/H	1892	50	2.6
16712644	Exon 1	A/T	1497	69	4.6
16712643	Exon 1	V/I	50	3	6.0
16712577	Exon 1	G/R	368	30	8.2
16712568	Exon 1	A/S	279	33	11.8
16712556	Exon 1	L/F	308	31	10.1
16712299	Exon 2	T/M	972	73	7.5
16710996	Exon 4	Y/C	106	0	0.0
16709389	Exon 5	P/S	324	20	6.2
16709366	Exon 5	M/I	360	27	7.5
16709364	Exon 5	Q/R	2402	0	0.0
16707979	Exon 7	H/Y	2418	42	1.7
16707352	Exon 9	F/L	5	0	0.0
16707265	Exon 9	L/N	1	0	0.0
16707259	Exon 9	M/V	2084	64	3.1
16706845	Exon 10	V/A	1684	84	5.0
16706839	Exon 10	K/T	1695	79	4.7
16706832	Exon 10	S/R	2006	60	3.0

Supplementary Table 3. Nucleotide diversity (**π**) and genetic diversity (Watterson's Theta θ_W) of *OsGDA1* in wild and cultivated rice accessions.

	O. rufipogon (n=53)		*indica* (n=44)		*japonica* (n=24)		*aus* (n=16)		*aromatic* (n=5)	
	θ_W	**π**	θ	**π**	θ_W	**π**	θ_W	**π**	θ_W	**π**
Total	0.0259	0.0340	0.0220	0.0329	0.0202	0.0314	0.0233	0.03262	0.02680	0.0321
Silent	0.0301	0.0399	0.0257	0.0392	0.0238	0.0368	0.0275	0.03872	0.03152	0.0377
Synonymous	0.0318	0.0468	0.0298	0.0366	0.0292	0.0458	0.0328	0.03986	0.04103	0.0501
Non-synonymous	0.0067	0.0072	0.0049	0.0042	0.0039	0.0067	0.0044	0.00492	0.00530	0.0066

EASE-MM Clade 1

Accession	Mutation	ddG	Class*	rASA	DIM Class
8502-Trop_jap-US	/	/	/	/	/
SRX621477-nivar	D379N	0.14	N	0.68	O → O
SRX621477-nivar	M244I	-0.18	N	0.22	O → D
SRX621481-nivar	A304S	-1.09	D	0.07	O → O
SRX621481-nivar	A95T	-0.45	N	0.48	O → O
SRX621481-nivar	D379N	0.14	N	0.68	O → O
SRX621481-nivar	I306V	-0.74	LD	0.06	O → O
SRX621481-nivar	M300L	0.32	N	0.26	O → O
SRX621481-nivar	R454S	-0.62	LD	0.63	O → D
11741-Aus-Sri_Lanka	A6V	0.18	N	0.67	O → D
11741-Aus-Sri_Lanka	A95T	-0.45	N	0.48	O → O
11741-Aus-Sri_Lanka	D379N	0.14	N	0.68	O → O
11741-Aus-Sri_Lanka	G56R	-0.49	N	0.77	O → O
11741-Aus-Sri_Lanka	M244I	-0.18	N	0.22	O → D
11741-Aus-Sri_Lanka	P237S	0.11	N	0.45	O → D
11741-Aus-Sri_Lanka	Y139C	-1.67	D	0.24	O → D
9917-Indica-Sri_Lanka	A6V	0.18	N	0.67	O → D
9917-Indica-Sri_Lanka	A95T	-0.45	N	0.48	O → O
9917-Indica-Sri_Lanka	G56R	-0.49	N	0.77	O → O
9917-Indica-Sri_Lanka	I67L	-0.79	LD	0.06	O → O
9917-Indica-Sri_Lanka	M244I	-0.18	N	0.22	O → D
9917-Indica-Sri_Lanka	M300L	0.32	N	0.26	O → O
9917-Indica-Sri_Lanka	P237S	0.11	N	0.45	O → D
ERX046640-rufip	A8V	-0.06	N	0.58	O → O
ERX046640-rufip	A95T	-0.45	N	0.48	O → O
ERX046640-rufip	D379N	0.14	N	0.68	O → O
ERX046640-rufip	V7A	-0.28	N	0.58	O → O

EASE-MM Clade 2

Accession	Mutation	ddG	Class*	rASA	DIM Class
11630-Basmati-Nepal	A304S	-1.09	D	0.07	O → O
11630-Basmati-Nepal	A59S	-0.53	LD	0.26	O → O
11630-Basmati-Nepal	A95T	-0.45	N	0.48	O → O
11630-Basmati-Nepal	I306V	-0.74	LD	0.06	O → O
11630-Basmati-Nepal	M300L	0.32	N	0.26	O → O
11630-Basmati-Nepal	R454S	-0.62	LD	0.63	O → D
8665-Temp_jap-US	A304S	-1.09	D	0.07	O → O
8665-Temp_jap-US	A59S	-0.53	LD	0.26	O → O
8665-Temp_jap-US	A95T	-0.45	N	0.48	O → O
8665-Temp_jap-US	D379N	0.14	N	0.68	O → O
8665-Temp_jap-US	I306V	-0.74	LD	0.06	O → O
8665-Temp_jap-US	M300L	0.32	N	0.26	O → O
8665-Temp_jap-US	R454S	-0.62	LD	0.63	O → D
9637-Indica-Vietnam	A304S	-1.09	D	0.07	O → O
9637-Indica-Vietnam	A59S	-0.53	LD	0.26	O → O
9637-Indica-Vietnam	D379N	0.14	N	0.68	O → O
9637-Indica-Vietnam	M300L	0.32	N	0.26	O → O
9637-Indica-Vietnam	R454S	-0.62	LD	0.63	O → D
SRX621480-nivar	A304S	-1.09	D	0.07	O → O
SRX621480-nivar	A59S	-0.53	LD	0.26	O → O
SRX621480-nivar	A95T	-0.45	N	0.48	O → O
SRX621480-nivar	D379N	0.14	N	0.68	O → O
SRX621480-nivar	I306V	-0.74	LD	0.06	O → O
SRX621480-nivar	M300L	0.32	N	0.26	O → O
SRX621480-nivar	R454S	-0.62	LD	0.63	O → D
11482-Aus-Bangladesh	A95T	-0.45	N	0.48	O → O
11482-Aus-Bangladesh	D379N	0.14	N	0.68	O → O
11482-Aus-Bangladesh	I306V	-0.74	LD	0.06	O → O
11482-Aus-Bangladesh	M300L	0.32	N	0.26	O → O
11482-Aus-Bangladesh	R454S	-0.62	LD	0.63	O → D
SRX367233-rufip	I306V	-0.74	LD	0.06	O → O
SRX367233-rufip	M300L	0.32	N	0.26	O → O
SRX367233-rufip	R454S	-0.62	LD	0.63	O → D

EASE MM Clade 3

Accession	Mutation	ddG	Class*	rASA	DIM Class
11453-Aus-India	A95T	-0.45	N	0.48	O → O
11453-Aus-India	D379N	0.14	N	0.68	O → O
11453-Aus-India	E450G	-1.43	D	0.39	O → D
11453-Aus-India	I452R	-0.70	LD	0.12	O → D
11453-Aus-India	R454S	-0.62	LD	0.63	O → D
11453-Aus-India	T109M	-1.26	D	0.33	O → D
8173-Temp_jap-US	A95T	-0.45	N	0.48	O → O
8173-Temp_jap-US	D379N	0.14	N	0.68	O → O
8173-Temp_jap-US	E450G	-1.43	D	0.39	O → D
8173-Temp_jap-US	I452R	-0.70	LD	0.12	O → D
8173-Temp_jap-US	L69F	-0.19	N	0.02	O → D
8173-Temp_jap-US	R454S	-0.62	LD	0.63	O → D
8173-Temp_jap-US	T109M	-1.26	D	0.33	O → D
9470-Trop_jap-Indonesia	A95T	-0.45	N	0.48	O → O
9470-Trop_jap-Indonesia	D379N	0.14	N	0.68	O → O
9470-Trop_jap-Indonesia	E450G	-1.43	D	0.39	O → D
9470-Trop_jap-Indonesia	I452R	-0.70	LD	0.12	O → D
9470-Trop_jap-Indonesia	L69F	-0.19	N	0.02	O → D
9470-Trop_jap-Indonesia	R454S	-0.62	LD	0.63	O → D
9682-Basmati-Bangladesh	A95T	-0.45	N	0.48	O → O
9682-Basmati-Bangladesh	D379N	0.14	N	0.68	O → O
9682-Basmati-Bangladesh	E450G	-1.43	D	0.39	O → D
9682-Basmati-Bangladesh	I452R	-0.70	LD	0.12	O → D
9682-Basmati-Bangladesh	L69F	-0.19	N	0.02	O → D
9682-Basmati-Bangladesh	R454S	-0.62	LD	0.63	O → D
SRX367229-rufip	A95T	-0.45	N	0.48	O → O
SRX367229-rufip	D379N	0.14	N	0.68	O → O
SRX367229-rufip	E450G	-1.43	D	0.39	O → D
SRX367229-rufip	I452R	-0.70	LD	0.12	O → D
SRX367229-rufip	R454S	-0.62	LD	0.63	O → D
SRX367229-rufip	T109M	-1.26	D	0.33	O → D
11646-Indica-India	A95T	-0.45	N	0.48	O → O
11646-Indica-India	D379N	0.14	N	0.68	O → O
11646-Indica-India	I452R	-0.70	LD	0.12	O → D
11646-Indica-India	L69F	-0.19	N	0.02	O → D
11646-Indica-India	R454S	-0.62	LD	0.63	O → D
11646-Indica-India	T109M	-1.26	D	0.33	O → D

Supplementary Table 4. Protein destabilization due to amino acid changes. Data in red font indicates highly destabilizing mutations due to change in Gibbs free energy (ddG) or due to potential change from ordered (O) to disordered (D) structure. Class* represent N: neutral, LD: likely destabilizing and D: destabilizing. rASA: relative accessible surface area. DIM: Disorder inducing mutation.

Rice NAT LOC_ID	Arabidopsis Ortholog	Rice Plant	Leaf		Root	
			RQM	sd	RQM	sd
LOC_Os01g55500	At2G34190	KO	1.12	0.2	3.5	0.1
		WT	1	0.2	1	0.1
		Van	0.47	0.2	1.8	0.2
		481-B	0.43	0.7	1.2	0.2
LOC_Os07g30810	At4G380590	KO	0.88	0.1	3.7	0.2
		WT	1	0.2	1	0.07
		Van	0.55	0.4	1.8	0.4
		481-B	0.5	0.4	0.77	0.3
LOC_Os09g15170	At2G34190	KO	0.65	0.1	2.8	0.1
		WT	1	0.1	1	0.1
		Van	0.2	0.2	1.8	0.4
		481-B	0.1	0.2	1.5	0.04

Supplementary Table 5: qRT-PCR results for three rice NATs most closely related to the two Arabidopsis xanthine transporter/permeases (At2G34190 and At4G380590). In general the NATS were not substantially differentially expressed in the leaves but in the KO line and Vandana they exhibited increased expression of the NATs

Supplementary **Table 6. List of p**rimers used for cloning and QRT-PCR

Name of the primer	Sequence (5’-3’)
qAMI4_F	GCTGCACAGAAACTGAATACTGG
qAMI4_R	TCCCTTAGTCTGCATTACCAGC
q61810_F2	GGTCATCAGGAAGCCGTACA
q61810_R2	ACCTCCATGGCCACAATCTAC
Ami_pGEX_F	GTCGGATCCATGGACGGCGGCTTCC
Ami_pGEX_R	CGCGCTCGAGTCACAAGTAGTTCATC
T-LOC_Os01g55500	TTCGTGGTCCCGATAATGGC
	GAAGGAACCTCTCGTGGTCG
T-LOC_Os09g15170	CCTACCATCATTGGGGGCTC
	CAAGCGATGGGTCACGGATA
T-LOC_Os08g28170	ACCTCTCTGCTCACCCAGAT
	GATCTGCGCTGAATGTTGGC
T-LOC_Os07g30810	CAACGGGCTAAACTTTGCGG
	GATCAAGGCGACGAGGAGAG
D-LOC_Os01g55500	CACGCTGGAGGTGAAAAACG
	CTTGAACGACCGGAACGGTA
D-LOC_Os07g30810	GAAGAGCCTTGCAGTTTGGC
	CAGGAATGGAGGCAAGGAGG
D-LOC_Os08g28170	CACCCCCACCACCATATGTC
	TGGAGCCAGTTCCAGTACCA
D-LOC_Os09g15170	ATGTCCGGCATGTTCCAGTT
	ACTTGCTGTGAGGATGTGGG
